# Recent advances in electrolyte molecular design for alkali metal batteries

**DOI:** 10.1039/d3sc06650a

**Published:** 2024-02-06

**Authors:** Digen Ruan, Zhuangzhuang Cui, Jiajia Fan, Dazhuang Wang, Yiying Wu, Xiaodi Ren

**Affiliations:** a Hefei National Research Center for Physical Sciences at the Microscale, CAS Key Laboratory of Materials for Energy Conversion, Department of Materials Science and Engineering, University of Science and Technology of China Hefei Anhui 230026 China xdren@ustc.edu.cn; b Department of Chemistry and Biochemistry, The Ohio State University Columbus OH 43210 USA wu@chemistry.ohio-state.edu

## Abstract

In response to societal developments and the growing demand for high-energy-density battery systems, alkali metal batteries (AMBs) have emerged as promising candidates for next-generation energy storage. Despite their high theoretical specific capacity and output voltage, AMBs face critical challenges related to high reactivity with electrolytes and unstable interphases. This review, from the perspective of electrolytes, analyzes AMB failure mechanisms, including interfacial side reactions, active materials loss, and metal dendrite growth. It then reviews recent advances in innovative electrolyte molecular designs, such as ether, ester, sulfone, sulfonamide, phosphate, and salt, aimed at overcoming the above-mentioned challenges. Finally, we propose the current molecular design principles and future promising directions that can help future precise electrolyte molecular design.

## Abstract 2

In response to societal developments and the growing demand for high-energy-density battery systems, alkali metal batteries (AMBs) have emerged as promising candidates for next-generation energy storage.

## Graphical abstract

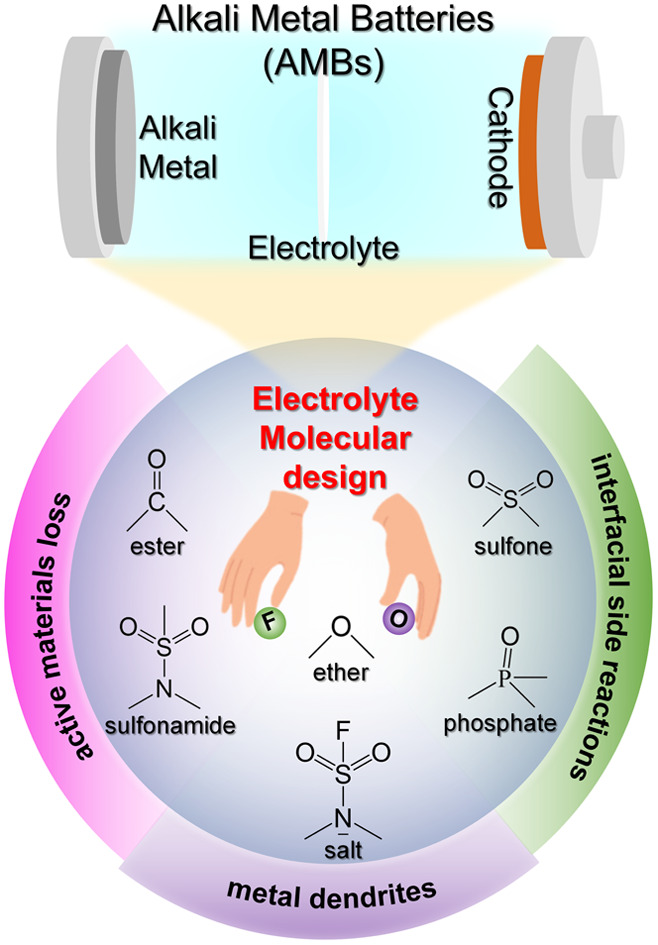

## 1. Introduction

Since their commercialization in the early 1990s, lithium-ion batteries (LIBs) have emerged as the predominant choice for energy conversion and storage in portable electronic devices and electric vehicles owing to their high energy density of almost 300 W h kg^−1^.^1–4^ However, the increasing demand for high-energy density batteries in recent years has posed challenges for traditional LIBs that utilize transition metal oxide cathodes and graphite anodes. These technologies appear to reach their limits in terms of energy storage capacity.^5–8^ As a potential solution, lithium (Li) metal has emerged as a promising candidate for alternative anode materials owing to its low electrode potential (−3.04 V *vs.* standard hydrogen electrodes) and high specific capacity (3860 mA h g^−1^),^7,9–12^ which have triggered the worldwide research on Li metal batteries (LMBs). Nonetheless, Li resources are limited in the earth's crust, comprising only 0.0017 wt%, and are unevenly distributed. In contrast, sodium (Na, 2.36 wt%) and potassium (K, 2.09 wt%) reserves are much more abundant and globally available.^13^ Na metal anode and K metal anode offer high theoretical capacities along with low redox potentials of −2.71 V and −2.93 V, respectively.^8,14,15^ Due to these characteristics, considerable efforts are underway to develop rechargeable Na metal batteries (NMBs) and K metal batteries (KMBs). These high-capacity alkali metal electrodes hold great promise for next generation high-energy batteries, potentially replacing conventional carbon-based anodes.^16,17^

**Fig. 1:**
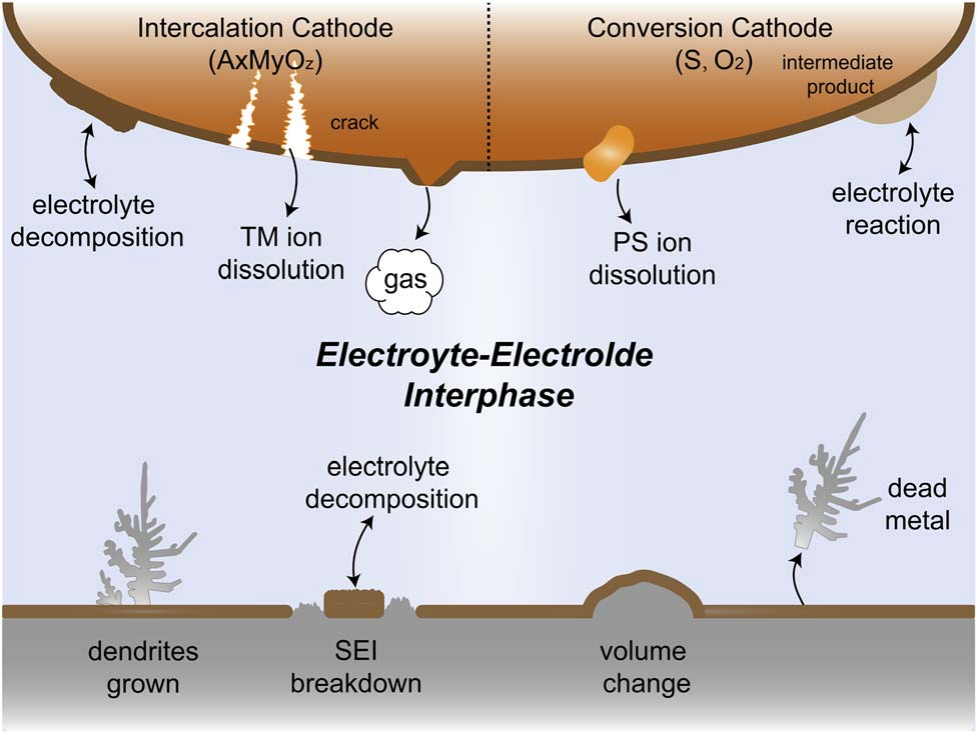
Schematic illustrations for the interfacial challenges of electrolyte and electrode in AMBs.

However, the practical application of alkali metal batteries (AMBs) still faces severe electrolyte–electrode interphase challenges, which greatly affect the reversibility and cycle stability of AMBs. As shown in Fig. 1, alkali metal ions tend to deposit non-uniformly on the substrate, leading to dendrite growth during continuous deposition/stripping. This becomes particularly problematic when using flammable electrolytes as these dendrites can spike the separator, causing short circuits in the battery. The resultant release of significant heat may lead to fire or even explosion, posing a significant safety hazard.^18^ In the Li metal system, the solid electrolyte interphase (SEI) receives a great spotlight due to its crucial role in the cycle life of the battery. Given the lowest electrochemical reduction potential of Li, during the initial Li deposition process, the electrolyte is preferentially reduced to form the SEI, blocking further contact between the electrolyte and Li. However, in the repetitive deposition and stripping processes, SEI fractures caused by the uneven expansion and contraction of Li metal lead to new surface exposure, resulting in the loss of active materials and the reduction in the coulombic efficiency (CE). Furthermore, during plating/stripping, the formed dendrites may break away from the substrate, resulting in the generation of dead metal.^19–21^ Despite their relatively higher electrochemical potentials compared to Li metal, Na and K metals are regarded to have higher reductive capability due to their lower ionization energy and electronegativity. Therefore, the challenge is even more forbidding to develop highly efficient electrolytes for Na and K metal batteries and take full advantage of their cost benefits.

The electrolyte and cathode also suffer from complex interfacial reactivity problems. AMBs can use a variety of materials as cathodes, such as commonly intercalation cathodes and conversion cathodes. Under extreme conditions such as high voltage and high temperature, the electrolyte is prone to oxidative decomposition, accompanied by gas production and dissolution of transition metal ions, which can cause cracking of the cathode material and cell capacity fading.^22–25^ Conversion cathodes like sulfur and oxygen with complex reaction processes can produce intermediates that may easily dissolve into or react with the electrolyte, which leads to active material loss and even cell failure.^26–29^ Therefore, addressing these challenges of electrolyte–electrode interphase is crucial for the successful application of practical AMBs.

It is noted that electrolyte as an important part of the battery serves a role that extends far beyond mere ion transportation. Its impact spans critical aspects such as interphase stability, electrochemical performance and safety.^30–32^ Commercially available electrolyte compositions have indeed played a significant role in driving battery development to date. However, as next-generation high-energy batteries continue to advance, they are encountering challenges that render conventional electrolytes insufficient to meet the growing demands. Consequently, a more profound and systematic investigation into the foundational aspects of electrolyte molecular design becomes imperative. By focusing on the molecular-level design of electrolytes, researchers can precisely tailor their properties and interactions. This precise molecular design of the electrolyte is expected to result in superior electrolyte properties and thus better electrochemical performance. The quest for more in-depth and fine-tuned electrolyte molecular design promises transformative breakthroughs. As we venture into this frontier, it is conceivable that new generations of batteries will emerge, reshaping the energy landscape and propelling us closer to a sustainable and electrified future. The pursuit of advanced electrolyte designs is undeniably a key pathway to overcoming the challenges faced by alkali metal batteries and fostering the continued advancement of battery technology.

This review begins with elucidating the primary mechanisms of interfacial failure observed in alkali metal batteries, encompassing highly reactive interphase instability, uncontrolled dendrite growth of the alkali metal anodes and severe electrolyte side reactions with cathode interphase or reaction intermediates. Subsequently, we delve into an extensive discussion of the recent advancements in the molecular design of various electrolyte components, such as new solvents based on ether, carbonate, sulfonamide, phosphate, co-solvents, and electrolyte salts. Emphasis is placed on the influence of these molecular designs on enhancing the interfacial stability for alkali metal batteries. Moving forward, the review of the diverse types of cathodes applied in alkali metal batteries, provides an in-depth examination of the electrolyte design for different cathode materials and their implications on battery performance.

In this review, we present the information and delineate the core principles of electrolyte molecular design in alkali metal batteries. We observe that current molecular designs predominantly focus on Li metal, with comparatively fewer studies dedicated to Na and K metal anodes. Given the chemical and physical similarities among alkali metals, many design principles applicable to Li metal electrolytes might also be relevant for Na and K metals. Consequently, this review emphasizes molecular designs in LMBs, with the aim of catalyzing further innovation in molecular engineering for Na and K metal electrolytes. Additionally, we will discuss the potential differences and specific considerations necessary for designing electrolytes across various alkali metal anodes.

## 2. Interfacial failure mechanisms in alkali metal batteries

The inherently high reactivity of alkali metal anodes inevitably leads to their interaction with solvent molecules and salts within the existing electrolyte, resulting in the formation of the SEI. The nature of this interfacial film largely determines the stability and reversibility of alkali metal anodes. In recent years, numerous studies have been carried out to solve the instability of different electrolyte components for the alkali metal anode. In this regard, organic carbonate and ether solvents, characterized by excellent electrochemical stability and high salt dissociation capability, have been extensively documented. In this section, we aim to explain the interfacial failure mechanisms in alkali metal batteries when using these prevalent solvents, with a particular focus on Li metal.

### 2.1 Carbonates

The commercialization of LIBs has been successful due to the use of lithium hexafluorophosphate (LiPF_6_) with a combination of cyclic ester ethylene carbonate (EC) and linear esters such as dimethyl carbonate (DMC), ethyl methyl carbonate (EMC), or diethyl carbonate (DEC) as the basic electrolyte formula.^32^ Cyclic carbonates, especially EC, are capable of forming the SEI on graphite, while the use of linear esters helps reduce electrolyte viscosity and enhance ion conductivity.^33,34^ On the cathode side, the –CO_3_– structure in carbonates effectively disperses lone pair electrons on the carbonyl oxygen, ensuring acceptable oxidation stability.

**Fig. 2:**
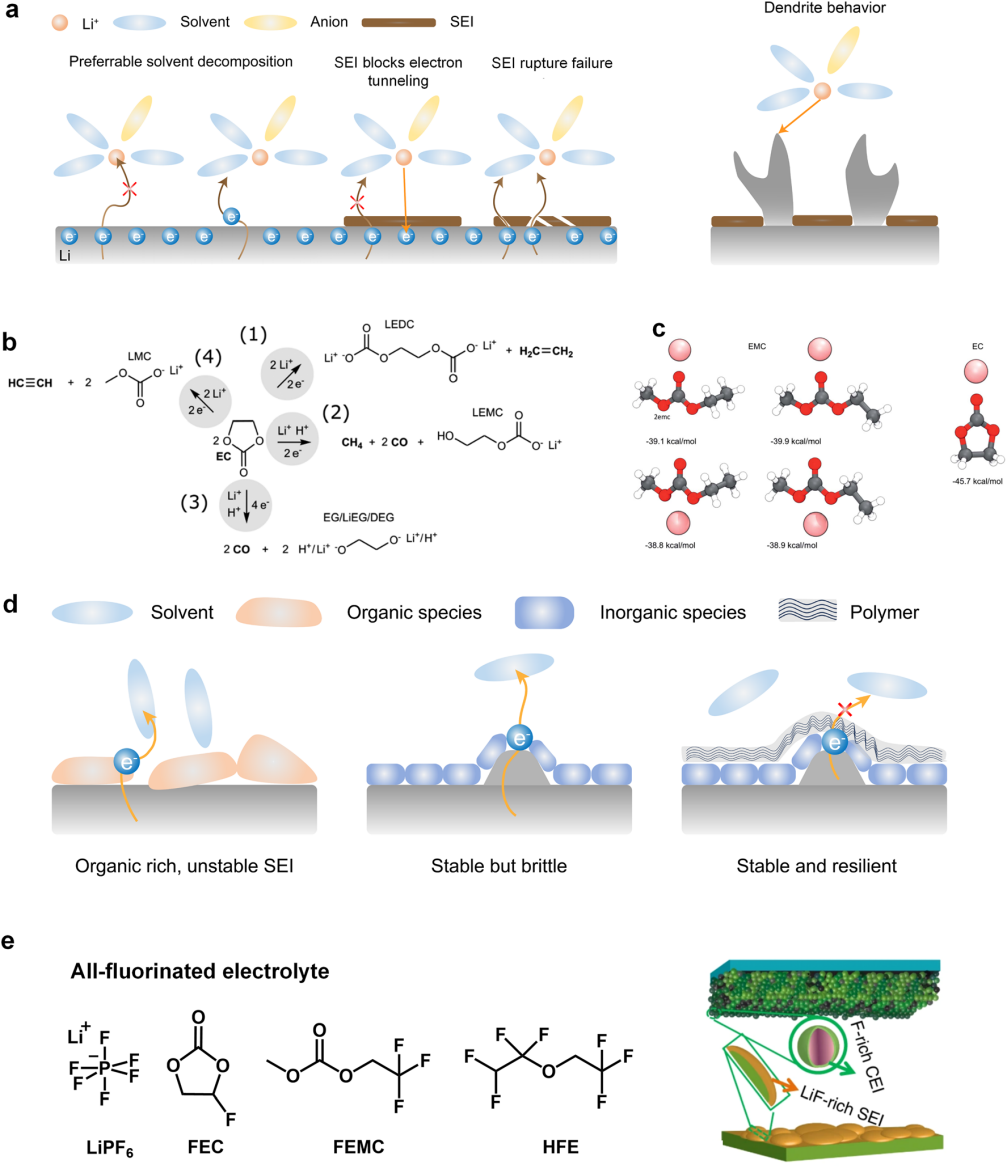
(a) Schematic diagram of electrolyte reduction priority and dendrite growth. (b) EC molecular reduction mechanism. Reproduced with permission. Copyright © 2020 American Chemical Society. (c) Binding energy comparison for Li-EC, Li-EMC. (d) Comparison of SEI dominated by organic, inorganic, and inorganic and polymer. (e) Composition and mechanism of action of all-fluorinated electrolyte. Reproduced with permission. Copyright © 2018, Nature Publishing Group.

However, when Li metal replaces graphite as the anode material, conventional carbonates fail to form an efficient SEI layer. This phenomenon arises from the inherently pronounced reactivity of carbonates with Li metal and the incapability of the generated SEI for effectively impeding the penetration of the electrolyte, thereby facilitating subsequent reactions (Fig. 2a). On the other hand, recent investigations have elucidated that the organic-rich SEI derived from commercial carbonate electrolytes (1 M LiPF_6_ in ethylene carbonate/diethyl carbonate (EC/DEC)) exhibit a heightened degree of swelling compared to those originating from four alternative electrolytes, namely, fluorinated carbonate additive, ether, high concentration electrolytes, and fluorinated ether solvent. During the charge transfer process, various organic carbonate solvents undergo electrochemical reduction to form side products. The reduction potential of carbonates typically falls within the range from 0.5 to 1 V, and it is widely accepted that EC undergoes preferential reduction in these carbonate blends to form the SEI.^35,36^ The reduction processes of EC can be diverse and varied, as depicted in Fig. 2b. Research by Aurbach *et al.*^37,38^ suggested that the reduction of cyclic carbonates tends to preferentially produce Li dialkylenecarbonates, while the reduction of linear carbonates tends to generate Li monoalkyl carbonates. When a mixture of cyclic and linear carbonates is present, such as EC and EMC, despite the higher content of EMC (2–3 times that of EC) in the electrolyte composition, the stronger binding energy of EC promotes its preferential coordination with Li ions (Fig. 2c).^23^ This means that more EC molecules participate in the solvation structure, increasing the likelihood of EC encountering electron transfer reactions associated with Li. Consequently, a greater amount of Li dialkylene carbonates is formed at the interface. The similarity between these organic groups and the electrolyte is high, which cannot prevent electrolyte penetration and further reactions. This means that the SEI generated by solvent reduction with dominant organic species is often inefficient, and continuous reduction reactions will still induce battery failure. The organic-dominated, scattered SEI proves to be ineffective in preventing electrolyte infiltration and inhibiting the sustained interfacial reactions, resulting in a diminished CE and persistent depletion of active anodes.

Additionally, the initially formed organic products continuously undergo complex decomposition, leading to the generation of Li_2_O or Li_2_CO_3_.^34,39^ For example, Xu *et al.* and Seo *et al.* demonstrated that Li alkyl carbonates have poor stability, which can readily convert to Li_2_CO_3_ induced by H_2_O or H^+^.^40,41^ Furthermore, investigations conducted by Campion and colleagues revealed that Li alkyl carbonates, exemplified by Li ethylene dicarbonate, undergo decomposition, yielding LiF, fluorophosphates, trimethylphosphates, carbon dioxide, and oligoethylene oxides when subjected to the influence of PF_5_.^42^ These inorganic compounds act as barriers for electronic transport and provide surface stability for organic Li salts. Consequently, the SEI exhibits a stratified structure, with the inner layer enriched with Li-stabilizing compounds and the outer layer accumulating incompletely reduced products. However, achieving complete reduction of the solvents is challenging as it involves multiple electron transfer reactions, resulting in very low coulombic efficiencies for Li‖Cu batteries using carbonate-based electrolytes throughout the cycle.^43,44^

It is worth mentioning that an increasing number of literature has proven that interface layers rich in inorganic products are crucial for highly efficient Li deposition and stripping as inorganic materials not only insulate electrons but also often have high interface energy that induces the lateral growth of Li deposition. Nevertheless, the augmented interfacial modulus poses challenges to the preservation of the SEI integrity during the subsequent deposition-stripping cycles.^45^ Conversely, in inorganic dominated SEI, the reduction of selected solvents, which can facilitate the introduction of peripheral oligomeric species, can fortify the structural integrity of SEI (Fig. 2d). However, the body of literature concerning the polymerization phenomena pertaining to cyclic carbonates remains relatively scant. In this regard, Tavassol *et al.*^46^ employed the advanced technique of matrix-assisted laser desorption time-of-flight mass spectrometry to investigate the SEI formed on different electrodes. The formation of SEI on Sn and Au electrodes was accompanied by the generation of a lengthy chain oligomer resulting from solvent decomposition. They found that the mechanism underlying oligomer formation involves radical initiation, followed by a propagation step. Additionally, it is noteworthy that not all polymers are beneficial for the Li deposition and stripping. Hang *et al.* investigated the effect of polymer dynamics on Li deposition behavior and concluded that the polymers as anodic coating with flowability or faster polymer dynamics exhibit higher CE.^47^

In recent years, the presence of highly insulating LiF within the SEI of high CE plating/stripping systems has garnered significant attention. LiF exhibits exceptional qualities as it effectively hinders electrolyte permeation, impedes electron transfer from Li metal, and maintains its stability without dissolving into the electrolyte.^38^ Achieving LiF enrichment in the interfacial layer can be realized through increasing salt concentration or the strategic fluorine substitution of carbonate esters (Fig. 2e).^48,49^ In 2008, Jeong *et al.*^50^ reported that the concentrated electrolyte (3.27 mol kg^−1^ LiN(SO_2_C_2_F_5_)_2_/PC) can inhibit Li dendrite growth. The author attributed the efficient deposition and stripping behavior to the enhanced decomposition of anions, and the subsequent studies increasingly targeted the enrichment of inorganic substances at the interface of high concentration electrolyte. For example, Chen *et al.*^51^ reported that the electrolyte formulation with 1.2 M lithium bis(fluorosulfonyl)imide in a mixture of dimethyl carbonate/bis(2,2,2-trifluoroethyl)ether (1 : 2 by mol) can enable the dendrite-free cycling of Li metal anodes with high CE (99.5%). On the other hand, in addition to the decomposition of anions, the utilization of fluorinated solvents also contributes to an increase in the content of inorganic species at the interface. Fan *et al.*^52^ have made remarkable strides in the efficient Li plating/stripping through the implementation of all-fluorinated electrolytes. After fluorination, the LUMO energy levels of carbonates are further lowered, making them more prone to reduction at the anode. However, unlike conventional carbonates, fluorinated carbonates (such as FEC and FEMC) are more likely to decompose on the surface of Li metal to produce inorganic LiF, which has high surface energy and low electronic conductivity, thereby inhibiting the continuous reduction of the electrolyte. The electrolyte formulated by their design (1 M LiPF_6_ in FEC/FEMC/HFE) exhibits stable Li plating/stripping for over 500 cycles in Li‖Cu batteries, with an average CE exceeding 99.2%.

### 2.2 Ethers

Ether-based electrolytes are renowned for their high reduction stability on alkali metals.^53,54^ At the beginning, considering the unsatisfactory cycling efficiency linked with carbonate-based electrolyte on Li metal, researchers have put their focus on ethers in pursuit of optimized deposition morphology and enhanced Li CE.^32^ Early research by Aurbach *et al.*^55^ demonstrated that linear ethers still react with lithium metal to generate lithium alkyl carbonate, but the reactivity is significantly weaker compared to cyclic ethers and carbonates. Therefore, the passivation layer formed on the Li metal in electrolytes dominated by linear ethers mainly originates from anions and a small amount of impurities. However, the conclusion drawn by Aurbach *et al.* is that the rough passivation layer formed as the anion cannot effectively protect the Li metal. Indeed, in many early works, prolonged cycling (>100 cycles) using ether electrolytes with LiPF_6_, LiBF_4_, LiAsF_6_ and LiOTF was challenging to achieve.^56–58^ From the current perspective, this is because these anions exhibit weak electrochemical reactivity towards the Li metal and the reaction products have limited protection capabilities. The emergence of LiFSI with strong interfacial passivation capability has significantly changed this situation. The easily reducible S–F bonds can generate abundant electron-insulating inorganic compounds such as LiF at the interface, contributing positively to the stable cycling of the Li anode. Therefore, the combination of LiFSI with ether-based electrolytes has become one of the mainstream approaches in current lithium metal batteries. Please note that the FSI^−^ anion might not be the universal solution to the alkali metal problem. Seh *et al.* reported that highly reversible Na metal with an extremely high CE of 99.9% could be achieved in 1 M NaPF_6_/glyme electrolyte.^59^ The excellent electrochemical performance was attributed to the NaF and Na_2_O found in the hybrid SEI. Furthermore, Doi *et al.* reported an F-free electrolyte, 0.1 M Na tetraphenylborate (NaBPh_4_) in DME, which enables a very high average Na CE of 99.85%.^60^ Despite containing only C, O, and Na elements, the SEI layer shows high stability and low resistance. Nevertheless, when similar electrolyte formulations were adapted in K metal batteries, significantly decreased stabilities were observed,^61^ which suggests that there are potentially significant differences between different alkali metal anodes in terms of interfacial stabilities.

In most cases, low-concentration ether-based electrolytes still fall short of forming a robust passivation layer on Li metal. For instance, employing 1 M LiFSI in DME electrolyte for Li‖Cu cells yields a CE below 90%, primarily attributed to Li consumption resulting from solvent reduction.^54,62^ As can be seen, the stable cycling of Li metal critically depends on highly reactive fluorinated solvents or anions *via* electrolyte engineering. In addition to the problem with the Li metal anode, ethers face more serious challenges in anodic oxidation stability. In contrast to carbonates, wherein the lone-pair electrons of the ethereal oxygen undergo additional conjugation with the 
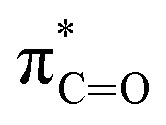
 antibonding orbital, resulting in the formation of a lower-energy filled orbital that makes electron loss more challenging, ethers exhibit a susceptibility to oxidation arising from the absence of phase-matched and energy-comparable empty orbitals capable of stabilizing the nonbonding electrons of oxygen.^63,64^ According to the report by Jang *et al.*,^65^ ether molecules could be oxidized to release protons and corrode the cathode when the voltage exceeds 4 V. This leads to the gradual dissolution of cathode materials and the loss of electrical contact with the surrounding conductive carbon, which results in rapid cell failure. Therefore, they cannot be directly used in combination with the cathodes that operate at voltages above 4 V. On the other hand, similar to the anode, the cathode electrolyte interface (CEI) layer formed from solvent decomposition fails to effectively isolate the electrolyte, leading to continuous side reactions and the accumulation of numerous by-products that impede Li^+^ transport. Consequently, Li depletion on the cathode interphase increases, leading to the sustained transformation of the electrode towards a rock-salt phase and ultimately causing the failure of the cathode.^66^

**Fig. 3:**
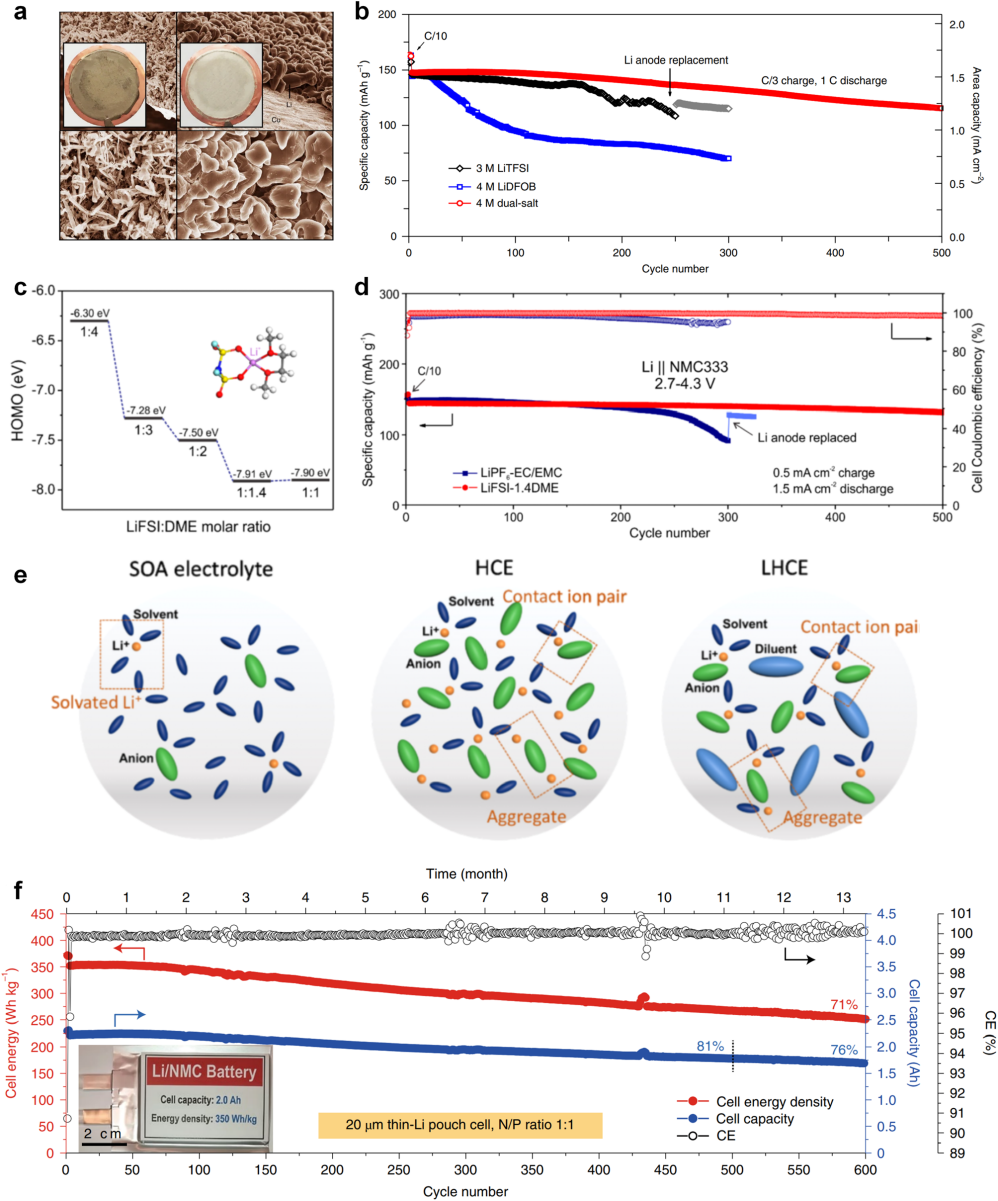
(a) Comparison of lithium deposition morphology. Reproduced with permission. Copyright © 2015, Nature Publishing Group. (b) Comparison of cycling performance of 4 M dual-salt, 4 M LiDFOB and 3 M LiTFSI. Reproduced with permission. Copyright © 2018, Nature Publishing Group. (c) HOMO energy levels with different LiFSI : DME molar ratio. (d) Comparison of cycling performance of HCE and LiPF_6_ in EC/EMC electrolyte. (e) Comparison of solvation structures of SOA electrolyte, high concentration electrolyte and local high concentration electrolyte. Reproduced with permission. Copyright © 2021, Nature Publishing Group. (f) Cyclic performance of pouch cell with 1.5 M LiFSI in DME/TTE electrolyte. Copyright © 2019 American Chemical Society.

The issues faced by ether-based electrolytes in relation to the anode and cathode can be addressed by increasing the coordination of anions within the solvent structure. High-concentration electrolytes enable increased anion incorporation into the solvation shell, thereby enhancing the proportion of anionic decomposition products within the SEI and CEI, simultaneously. In 2011, Watanabe *et al.*^67^ found that the ether-based electrolyte with an equimolar ratio of LiTFSI-glyme shows an oxidation stabilization potential of more than 5 V at the platinum electrode and achieves the cycling of high voltage LCO cathode under 4.2 V. Unfortunately, the cell suffered severe capacity degradation due to the continuous electrolyte decomposition. In 2013, Suo *et al.*^68^ presented a ‘Solvent-in-Salt’ electrolyte characterized by an exceptionally high salt concentration (7 M LiTFSI in DME/DOL; 1 : 1 by volume) and an elevated Li^+^ transference number. This formulation proves effective in mitigating Li dendrite growth and morphological changes, consequently improving the cyclic and safety performance. In addition, Qian and co-workers^69^ conducted a study on the efficacy of a highly concentrated ether electrolyte system (4 M LiFSI in DME) in enhancing the CE and deposition morphology of Li metal. As depicted in Fig. 3a, the Li deposition in this electrolyte demonstrates a dense, large-granular growth with a bright, silver-white metallic film. This stands in stark contrast to the dark gray, dendritic growth observed in traditional low-concentration electrolytes. Furthermore, Jiao and co-workers^70^ reported a dual-salt HCE consisting of 2 M LiDFOB (lithium bis(fluorosulfonyl)imideborate) + 2 M LiTFSI-DME electrolyte. The oxidative decomposition of the dual-salt components enriched the CEI with boron (B) and fluorine (F) elements, which are highly effective in protecting the cathode. As shown in Fig. 3b, the dual-salt high-concentration ether-based electrolyte greatly extended the cycle life of a 4.3 V-class Li‖NMC111 (LiNi_0.33_Co_0.33_Mn_0.33_O_2_) cell, with a capacity retention rate of ∼80% after 500 cycles of reversible cycling. Ren *et al.*^71^ investigated the oxidation stability and cycling performance of LiFSI at different concentrations in DME. The results showed that as the coordination number of anions in the solvent structure increased, the HOMO energy level of the electrolyte decreased to show higher oxidation stability (Fig. 3c). Furthermore, applying high concentration of LiFSI with labile S–F bond promotes interfacial LiF enrichment and effectively stabilizes both cathode and anode interphases. This ether-based electrolyte significantly improved the cycling stability of high voltage LMBs for over 500 cycles (Fig. 3d). Notably, compared to solvent anions, these high-concentration electrolytes foster more comprehensive decomposition and yield inorganic compounds such as LiF, Li_3_N, Li_2_O and Li_2_CO_3_, characterized by low solubility and rapid Li metal passivation capabilities. This electrolyte approach has also been applied to Na and K metal anode, where greatly improved cycling stabilities and CEs were obtained with high concentration of FSI^−^ anion in the electrolytes.^72,73^ However, challenges persist in the application of high-concentration electrolytes, including elevated viscosity and cost considerations.^74^

Recently, the researchers at Pacific Northwest National Laboratory (PNNL) have proposed the concept of localized high-concentration electrolyte (LHCE) structures, incorporating low-polarity, non-coordinating hydrofluorinated ethers to dilute high-concentration electrolytes and attain highly concentrated solvation structures at practical low-concentration regimes (Fig. 3e).^75^ The adoption of LHCEs significantly extends the lifespan of Li metal batteries and propels their advancement. Fig. 3e compares the solvation structures of low-concentration, high-concentration, and localized high-concentration electrolytes.^51^ Impressively, Niu *et al.*^76^ achieved stable cycling of a 1 Ah pouch cell for over 600 cycles with the use of advanced LHCE, reaching a remarkable energy density exceeding 400 W h kg^−1^ under appropriate pressure. This achievement represents one of the best reported values for practical Li metal batteries in the literature (Fig. 3f). Nevertheless, the current state-of-the-art localized high-concentration structures face some limitations, including limited cycling life due to rapid anion consumption and the adverse effects of excessive anion coordination, particularly at the anode interphase. On the other hand, excessive anionic coordination can lead to strong interfacial reactivity under elevated temperature conditions, thereby resulting in potential safety hazards.^77^

The structural design of ether molecules is also an effective strategy to enhance the stability of ethers at the cathode and anode interphases. Representatively, Yu *et al.*^78^ synthesized fluorinated 1,4-dimethoxylbutane (FDMB) based on DMB and studied the effect of fluoride on the molecular properties. In comparison to DMB, fluorinated FDMB demonstrated superior cathode stability and lithium metal compatibility (CE of ∼99.52%). Furthermore, they adjusted the fluorination ratio of the basic structure of 1,2-diethoxyethane (DEE) to synthesize a series of compounds, including F3DEE, F4DEE, F5DEE, and F6DEE.^79^ Among them, F5DEE, incorporating both –CF_3_ and –CF_2_H moieties, exhibited optimal Li metal compatibility, achieving a Li CE of 99.9% over more than 400 cycles. This highlights molecular design as an effective electrolyte strategy to enhance the electrochemical performance of LMBs, which will be discussed in detail in the following sections.

### 2.3 Alkali metal dendrite growth

The growth of Li dendrites is closely associated with the formulation of the electrolyte and the components in the SEI. Essentially, the formation of uneven interfaces induced by the deposition behavior of the electrolyte leads to the primary cause of dendrite formation, which is the presence of non-uniform electric fields.^80^ The pointed ends of Li dendrites can penetrate the SEI film, leading to the exposure of fresh Li to the electrolyte and the subsequent formation of new SEI films. These repetitive processes not only consume organic electrolytes and Li metal but also degrade the cycling efficiency of LMBs. Consequently, the development of a stable SEI is of utmost importance. The deposition process of Li involves nucleation and growth, with the later stage of growth having a relatively low energy barrier, making it a more stable phase.^81^ To a large extent, the morphology of the deposited Li metal is determined by nucleation and early-stage growth processes. It should be noted that the formation of SEI precedes Li deposition, and the diffusion of Li ions through the SEI poses a higher diffusion barrier compared to the electrolyte. Therefore, the SEI induced by electrolyte components plays a crucial role in the nucleation and growth of Li metal.

**Fig. 4:**
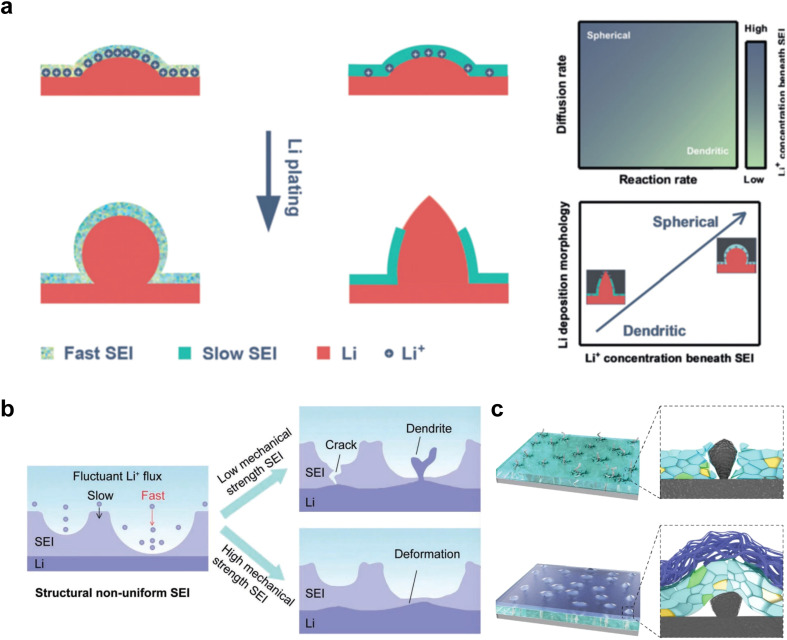
(a) The diffusion-reaction competition mechanism causing spherical/dendritic Li deposition with fast SEI/slow SEI. (b) Scheme of the morphology evolution in LMBs with a nonuniform SEI. (c) Cracks and distortions of SEI under low and high mechanical strength, respectively.

Chen *et al.*^82^ proposed a diffusion-reaction competition mechanism, in which they divided the behavior of Li deposition into two controlled stages: interfacial diffusion and deposition reaction. Their experimental results confirmed that the diffusion control of slow SEI is more prone to dendrite formation. This is because slow interfacial diffusion leads to a decrease in Li^+^ concentration below the SEI layer, and a small amount of Li tends to form dendritic morphologies (Fig. 4a). The formation of Li dendrites involves two processes: Li nucleation and Li growth. The growth process occurs immediately after nucleation, where the dendrites develop on the surface of nuclei and become incorporated into the Li metal lattice structure. The ultimate morphology of the deposited Li is strongly influenced by the nucleation and early growth stages. Understanding Li nucleation and early growth is crucial for exploring effective strategies to inhibit dendrite formation, ensuring the safety and long lifespan of LMBs. This result emphasizes the crucial role of enhancing Li^+^ diffusion within the SEI layer in Li deposition.

On the other hand, besides the ionic conductivity, the uniformity and mechanical toughness of SEI also play a crucial role in the deposition and stripping process. In particular, the structural non-uniformity of the SEI plays a disruptive role in the distribution of Li ion flux. This non-uniformity arises from variations in thickness and chemical composition induced by complex compounds present in the actual SEI.^83^ Furthermore, when the anode surface experiences fluctuations caused by the uneven distribution of Li ions and subsequent spot accumulation, SEI with low mechanical strength is prone to breakage.^84^ As a result, fresh Li metal is exposed through these cracks, creating an environment where both electrolyte decomposition and Li dendrite growth can competitively occur due to the thermodynamic activity of Li metal (Fig. 4b).

An SEI with sufficient mechanical strength plays a crucial role in inhibiting dendrite growth. Recently, Zhang and coworkers proposed the use of trioxane as an additive in LHCE to compensate for the lack of toughness in SEI.^45^ Trioxane decomposes prior to DME and allows the formation of a dual-layer SEI with an inner inorganic-rich layer and an outer polymer-rich layer at the interface. The designed electrolyte effectively extends the lifespan of LMBs.

### 2.4 Cathode interfacial stability

LiCoO_2_ (LCO), nickel-rich (NMC) and Li-rich manganese-based (LMR) layered oxides are reported to be highly promising cathodes for Li metal batteries due to their higher specific capacity and higher working potential.^66,85,86^ LCO has captured a significant share of the 3C electronics market due to its excellent packing density. However, the oxidation of Co^3+^ may lead to the loss of lattice oxygen because of the large overlap between the Co^3+/4+^ t_2g_ and O^2−^ 2p band. Therefore, the early LCO had a cut-off voltage of only 4.2 V and a capacity of 140 mA h g^−1^. In electrolyte engineering, electrolyte additives can regulate the composition of CEI to inhibit the occurrence of harmful phase transitions,^87,88^ thereby increasing the cut-off voltage of LCO. For example, Yan and co-workers^89^ demonstrate that the additive (potassium(4-methylsulfonylphenyl)trifluoroborate, SPTF) can inhibit the dehydrogenation reaction of EC. Also, a series of characterizations (*in situ* XRD, sXAS, AFM, HRTEM, NMR, cryo TEM) were used to demonstrate that trace additives not only regulate EC dehydrogenation but also construct more effective CEI (thin, sturdy, and stable). The modified CEI can suppress Co shuttling and effectively alleviate anode decay, ultimately achieving excellent performance in high-voltage LCO pouch cell. Recently, Zhang *et al.*^90^ used phytate lithium (PL) as a multifunctional additive to achieve high-voltage LCO. The remarkable chelating capability of PL with Co significantly enhances the structural stability, effectively preventing the detrimental H1-3 phase transition and Co dissolution. Furthermore, the robust CEI enabled by PL, along with its ability to annihilate oxygen radicals, contributes to the substantial suppression of parasitic interfacial side reactions.

Notedly, NMC is considered to be hopeful cathode materials for high energy density LMBs *via* increasing the voltage,^91^ whereas an excessively high cut-off charging voltage will cause a series of problems, such as irreversible phase changes, aggravated side reactions, and transition metal dissolution, which will cause premature capacity failure. Not only that, the inert components that were originally thought not to participate in the reaction, such as current collectors, binders, and conductive carbon, will also degrade at a sufficiently high charging voltage.^92^ To overcome these problems and extend the life of high-voltage LMBs, some interphase modification strategies can be used to effectively protect high-voltage NMC cathode. For instance, Cui *et al.*^93^ proposed that the use of semicrystalline Li niobate-coated layer can effectively improve the stability of low concentration ether-based electrolyte to cathode. Unlike traditional crystalline materials, semicrystalline Li niobate has higher ionic conductivity and better mechanical properties. Zhou *et al.*^94^ proved that the metal–organic framework (MOF)-based coating with sub-nano scale pore structures on the surface of NMC811 could successfully achieve an interfacial de-solvation process, which restricts the contact between the free ether molecules and cathodes, thus significantly enhancing the electrode stability in a diluted ether-based electrolyte. In addition, electrolyte engineering has been also widely adopted. Increasing the salt concentration^71,95^ and modifying the solvation structure^96,97^ have all made great progress, but the cathode stability is still largely limited by the intrinsic antioxidant ability of the individual electrolyte components. Furthermore, replacing NMC with LMR is one of the most effective methods to further increase battery energy density.^98^ However, a cut-off charging voltage of 4.8 V is unattainable for most ether-based electrolytes that are stable for Li metals. The LMR currently used for LMBs relies on fluorinated esters as only fluorinated esters have high stability at high voltage and can form a stable passivation layer on the negative electrode.

In addition to the above-mentioned poor oxidation stability of ether-based solvents incompatible with high-voltage cathode interphase, there are also significant cathode interphase stability challenges for other types of alkali metal batteries such as alkali metal–sulfur and alkali metal–air batteries. In alkali metal–sulfur batteries, the solid–liquid conversion reaction at the cathode interphase is closely related to the electrolyte components, which can markedly affect the reaction kinetics and the utilization of sulfur active material in the cell. In addition, the dissolution of polysulfides in the electrolyte can produce a “shuttle effect”-induced corrosion of the alkali metal anodes, leading to severe self-discharge and cycle capacity decay, while some insoluble and insulating sulfides can accumulate on the surface of alkali metal anodes.^27,99,100^ Similar to polysulfides, the dissolved O_2_ at the cathode of the alkali metal–air batteries can diffuse to the anodes and corrode the alkali metals.^29^ This is because high polarity and oxygen solubility are crucial for gas electrodes, but high polarity often has high reactivity for Li metals, making it difficult to achieve both high dissolved oxygen content and low negative electrode reactivity simultaneously. In the meantime, the cathode intermediates also are prone to react with the unstable electrolytes, especially the solvents, which hinders the reaction process of cathode and reduces the reaction reversibility.

## 3. Molecular design for the interface stability of alkali metal batteries

The performance failure of alkali metal batteries mainly results from the instability of the interphase between the electrodes and electrolyte. In previous studies, the modulation of electrolyte components is a direct and effective strategy to stabilize alkali metal anodes. However, the current electrolyte component formulation mainly relies on trial and error. Recently, the rational and precise molecular design of solvent and salt as well as the solvation structure of the electrolyte can effectively regulate the properties of the bulk electrolyte and SEI towards the extremely reactive alkali metal anodes and different cathodes including intercalation-type cathode (transition metal oxides) and conversion-type cathode (sulfur and air), facing the challenges of electrolyte decomposition under high-voltage and electrolyte interaction with intermediates, respectively. Strategies such as solvent fluorination and steric effect can optimize the ion–solvent interaction and adjust the interphase components, especially increasing the inorganic fluoride content, which helps to stabilize the electrolyte/electrode interphase and inhibit dendrite growth. Additionally, optimum molecular design can regulate the polarity and molecular orbital energy levels of the electrolyte molecules, thereby reducing the side reactions between the electrolyte and electrode. In this section, the molecular designs of electrolyte components for alkali metal batteries are summarized.

### 3.1 Molecular design for high-voltage alkali metal batteries

Alkali metals are extremely favorable anode materials with low electrode potential and high specific capacity.^29,73^ Compared with high-voltage cathodes, the batteries can provide high output voltage and capacity, which enables the realization of high energy density battery systems. In this section, we will focus on the novel molecules emerging in the electrolytes of high-voltage alkali metal batteries, in particular, Li metal batteries, and analyze their design strategies and working mechanisms for stabilizing electrode–electrolyte interphases.

#### 3.1.1 Ether solvent design

Due to the reduction stability of ether-based solvents, they are commonly used in alkali metal batteries to mitigate the reaction between the solvents and highly reactive anodes. However, the poor oxidation stability of ether-based molecules limits their employment in high-voltage cathodes and therefore affects the electrochemical stability of high-voltage alkali metal batteries.^30,70,71,101,102^ It is particularly important to investigate and design different ether molecular structures to improve the compatibility of ether-based solvents with high-voltage cathodes and further maintain stability with alkali metal anodes.

##### 3.1.1.1. Non-functionalized ethers

Non-functionalized ether molecules usually refer to molecules that do not contain other functional atoms such as fluorine or other atoms. Ether solvents, such as DME, usually have good compatibility with alkali metals and do not react violently with alkali metal electrodes, and thus are widely used in alkali metal cells.^71,103^

**Fig. 5:**
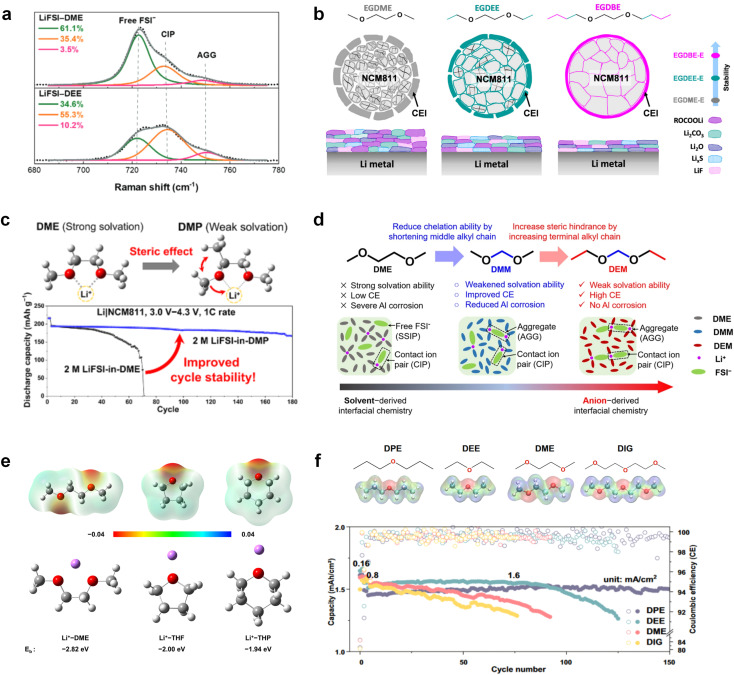
Non-functional substituted ether solvents show weak coordination with Li^+^. (a) Raman spectra and the population of free FSI^−^ (green line), CIPs (orange line), and AGGs (pink line) in the LiFSI-DME and LiFSI-DEE electrolyte systems. Reproduced with permission. Copyright © 2021 WILEY-VCH. (b) Illustration of the effects of EGDME-E, EGDEE-E, and EGDBE-E on the interfacial property of NMC811 cathode and Li metal anode. Reproduced with permission. Copyright © 2023 WILEY-VCH. (c) Solvent molecular design methodology of DEM for tailoring electrolyte solvation to promote the formation of an anion-dominated solvation structure in 1 m LiFSI/DEM. Reproduced with permission. Copyright © 2022 American Chemical Society. (d) Schematic illustration of steric hindrance and interaction with Li^+^ and the calculated minimum electrostatic potential (ESP) for DME and DMP. Reproduced with permission. Copyright © 2022 American Chemical Society. (e) Electrostatic potential mappings of DME, THF, and THP and the corresponding binding energies of Li^+^-solvent coordination structures. Reproduced with permission. Copyright © 2023 American Chemical Society. (f) The ESP maps of the studied ether molecules and their long-term cycling performance of Li‖NMC811 coin cells at 1.6 mA h cm^−2^. Reproduced with permission. Copyright © 2023, Nature Publishing Group.

Meanwhile, to address the cathodic stability issue of non-functionalized ether molecules, researchers have explored various molecular designs to regulate the solvation structure and interphase compositions. Weakly solvating ether solvents obtained by molecular design is a popular method to solve the above problems in recent years. The weakly solvating molecules can promote the formation of contact ion pairs (CIPs) and cation–anion aggregates (AGGs) in the electrolyte and induce the formation of dense interphases on both the cathode and anode, which can not only stabilize the electrode and inhibit the constant side reactions between the electrode and the electrolyte but also inhibit the growth of alkali metal dendrites and induce the uniform deposition of alkali metals. Chen *et al.*^19^ designed 1,2-diethoxyethane (DEE) by extending the terminal alkyl chain length of DME molecules (Fig. 5a). Wang *et al.*^104^ also extended the terminal alkyl chain length of DME molecules to design ethylene glycol dimethyl ether (EGDBE) to reduce the solvation ability of DME (Fig. 5b). The extension of the terminal alkyl chain length can improve the comprehensive stability of ether electrolytes and the weakly solvating structure can also be achieved at the same time. Ma *et al.*^105^ also modulated the steric effect of the solvent structure by rationally designing the carbon chain. The design of dipropylene glycol dimethyl ether (DMM) greatly reduced the solvent biotoxicity and obtained a weakly solvating structure, and the generated inorganic interphases to effectively passivate the cathode and the anode of potassium ion batteries. In addition, based on the structure of DME, Ma *et al.*^106^ obtained a weakly solvating molecule dimethoxymethane (DMM) by shortening the internal carbon chain of DME, which achieves uniform Li deposition under a low temperature of 40 °C due to the low desolvation energy of DMM. Furthermore, Ding *et al.*^107^ designed diethoxymethane (DEM) by not only shortening the internal alkyl chain but also increasing the terminal alkyl chain of DME to achieve a weak solvation effect (Fig. 5c). Park *et al.*^108^ designed 1,2-dimethoxypropane (DMP) to modulate the electrolyte solvation structure by increasing the spatial effect (Fig. 5d). Zhang *et al.*^96^ designed cyclic tetrahydropyran (THP) to modulate the molecular solvation ability by the six-membered ring structure (Fig. 5e). Additionally, a monodentate ether dipropyl ether (DPE) was proposed by Li *et al.*^109^ A weakly solvating solvation structure was effectively achieved by reducing the number of oxygen atoms and increasing the molecular steric resistances in the ether molecule, which endured long-term cycling for practical LMBs under high voltage (4.3 V) operation (Fig. 5f). Similarly, utilizing the monoxo ether structure, Ma *et al.*^110^ designed butyl methyl ether (BME) to compose a dilute electrolyte with weak solvation capability. This electrolyte exhibits an anion-rich solvation structure that forms an excellent CEI on sulfurized polyacrylonitrile (SPAN), effectively preventing the dissolution of polysulfides into the electrolyte and avoiding the shuttle effect. At the same time, the anion-derived SEI promotes the fast Li^+^ plating kinetics. In designing solvent molecules, longer carbon chains do not necessarily result in a weak solvation effect. The key to adjusting the number of carbon atoms to obtain weak solvation is introducing a significant steric effect to decrease the coordination between cation and oxygen atoms. All in all, the common advantage of these solvation structures is the enrichment of CIPs and AGGs to enhance the anion reactivity. Thus, this molecular design enables the suppression of the electrode–electrolyte interaction and forms a thinner, denser, and more inorganic-rich interphase. However, weak solvation also brings about an increase in the free solvent content due to the weak coordination between the solvent and cation.

**Fig. 6:**
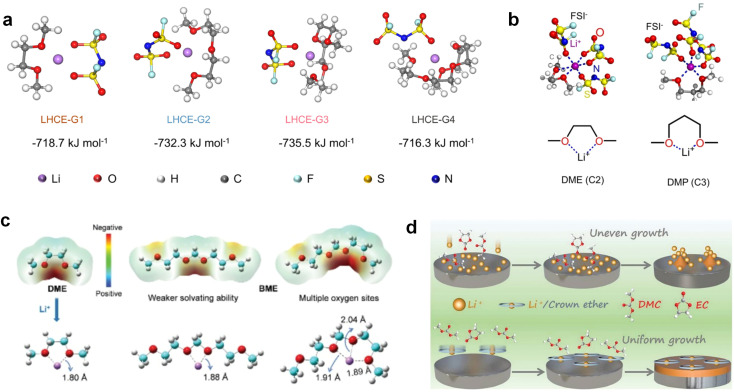
Non-functional substituted ether solvents showing strong coordination with Li ions. (a) Binding energies of different electrolyte complexes in LHCE-G1, LHCE-G2, LHCE-G3, and LHCE-G4. Reproduced with permission. Copyright © 2023 American Chemical Society. (b) Characterizations of ion solvation structures in C2-ELY and C3-ELY retrieved from MD simulations. Reproduced with permission. Copyright © 2023 WILEY-VCH. (c) ESP of DME and BME solvent and their optimized binding geometry with Li^+^. Reproduced with permission. Copyright © 2023 WILEY-VCH. (d) Schematic of the effect of crown ether additives on the decomposition process of solvents. Reproduced with permission. Copyright © 2020 WILEY-VCH.

Accordingly, strongly solvating solvents have been investigated with the aim of weakening the reactivity of free solvents at the interphase by increasing the salt concentration and enhancing the coordination of the solvent. Chen *et al.*^111^ proposed the concept that strong solvation effects can also enhance the oxidation stability. The triglyme (G3)-based electrolyte with the largest Li^+^ solvation energy among different linear ethers demonstrates greatly improved stability on Ni-rich cathodes under an ultrahigh voltage of 4.7 V (Fig. 6a). This is because ether electrolytes with a stronger Li^+^ solvating ability could greatly suppress deleterious oxidation side reactions by decreasing the lifetime of free labile ether molecules. Apart from increasing the number of the ethylene glycol monomer, Chen *et al.*^112^ also extended the length of the central alkyl chain. The designed 1,3-dimethoxypropane (DMP, C3) forms a unique six-membered chelating complex with Li^+^, whose stronger solvating ability suppresses oxidation side reactions (Fig. 6b). In addition, the favored hydrogen transfer reaction between C3 and anion induces a dramatic enrichment of LiF on the cathode surface. As a result, the C3-based electrolyte enables the greatly improved cycling of nickel-rich cathodes under 4.7 V. Wu *et al.*^113^ designed bis(2-methoxyethoxy)methane (BME) to form unique tridentate coordination by extending the ether chain length (Fig. 6c). Wang *et al.*^114^ used a strongly solvating 15-crown-5 (15-C-5) as an additive to induce the uniform deposition of Li^+^ to inhibit dendrite growth (Fig. 6d). However, the oxidation stabilities of these electrolytes need to be further investigated.

##### 3.1.1.2. Functionalized ether molecules

**Fig. 7:**
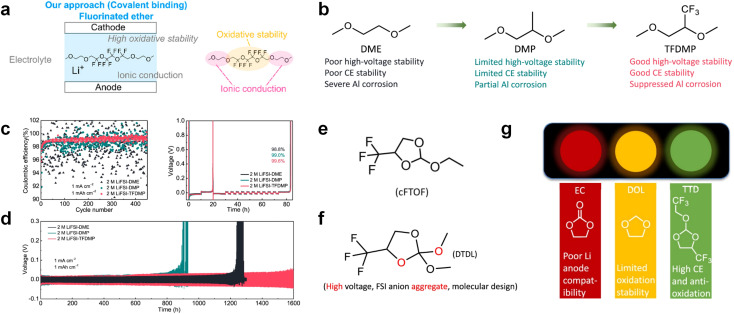
(a) Covalently attaching an ether to the hydrofluoroether allows for both high ionic conductivity and oxidation stability. Reproduced with permission. Copyright © 2020 American Chemical Society. (b) Solvent structure and associated properties of TFDMP. (c) CE test of Li‖Cu asymmetric cells using different electrolytes at 1 mA cm^−2^ with a cutoff capacity of 1 mA h cm^−2^. (d) Average CE test of three electrolytes by the Aurbach method. Reproduced with permission. Copyright © 2023, Nature Publishing Group. (e and f) Molecular structures of solvents, cFTOF and DTDL. Reproduced with permission. Copyright © 2022, Nature Publishing Group. Copyright © 2022 WILEY-VCH. (g) Design critical of TTD. Reproduced with permission. Copyright © 2022 American Chemical Society.

As the most electronegative element, fluorine is often used to modify ether molecules to enhance the oxidation stability, resulting from the reduced oxygen electron cloud density *via* the strong electron-drawing effect of fluorine atom. By varying the length and the type of ether group as well as the length of the fluorinated segment, Amanchukwu *et al.*^115^ synthesized and compared a new class of fluorinated ether electrolytes that combine the oxidation stability of hydrofluoric ether moiety with the ionic conductivity of ether moiety in a single compound (Fig. 7a). This demonstrates that both different fluorine/oxygen ratios and ether chain lengths can significantly affect the oxidation stability and ionic conductivity of fluorinated ethers. Besides that, they proposed that the fluorinated segment may orient toward the electrode surface, limiting ether accessibility to the surface. In addition, owing to the decreasing oxygen electron cloud density by fluorine, the interaction between Li^+^ and oxygen is weakened in the solvation structure, leading to a weak solvation effect. Recently, several works reported these weakly-solvating ether electrolytes (WSEE) and investigated the solvation behavior of electrolytes and their correlation with battery performance. In particular, the –CF_3_ group is often introduced into ether molecules (both cyclic and chain) to regulate the solvation ability because of its high fluorine content and common availability. Zhao *et al.*^116^ introduced the targeted trifluoromethylation of 1,2-dimethoxyethane to produce 1,1,1-trifluoro-2,3-dimethoxypropane (TFDMP), which weakened Li^+^-solvent interactions and led to the formation of CIPs in the solution (Fig. 7b). In the meantime, the oxidative stability of this designed ether-based molecule is also improved due to the introduction of the –CF_3_ group for reducing the electron cloud density of oxygen in the molecule. Hence, the 2 M LiFSI/TFDMP electrolyte exhibits up to 4.8 V oxidation potential measured by LSV and high Li CE of 99.6%, which enhances the cycle stability of the high-voltage practical LMBs (Fig. 7c and d). Additionally, some ring-chain ether molecule with –CF_3_ group designs^117–119^ such as 2-ethoxy-4-(trifluoromethyl)-1,3-dioxolane (cFTOF) and 2,2-dimethoxy-4-(trifluoromethyl)-1,3-dioxolane (DTDL) as well as 4-(trifluoromethyl)-1,3-dioxolane (TFDOL) can also enable high voltage stability and enhance Li^+^-FSI^−^ coordination to derive high LiF interphase (Fig. 7e and f). However, when the –CF_3_ introduction leads to a high F/O ratio, such fluorinated ethers are difficult to use as solvents due to the weak solvation ability. A fluorinated dioxolane-based cyclic molecule 2-(2,2,2-trifluoroethoxy)-4(trifluoromethyl)-1,3-dioxolane (TTD) with two –CF_3_ groups was developed as a co-solvent for DME (Fig. 7g). 1.5 M LiFSI-2DME-8TTD electrolyte exhibits remarkable oxidation stability up to 6 V and a high Li CE of 99.4% resulting from the efficient regulation of the electrolyte solvation structure and consequent anion-derived inorganic SEI layer formation.^120^

**Fig. 8:**
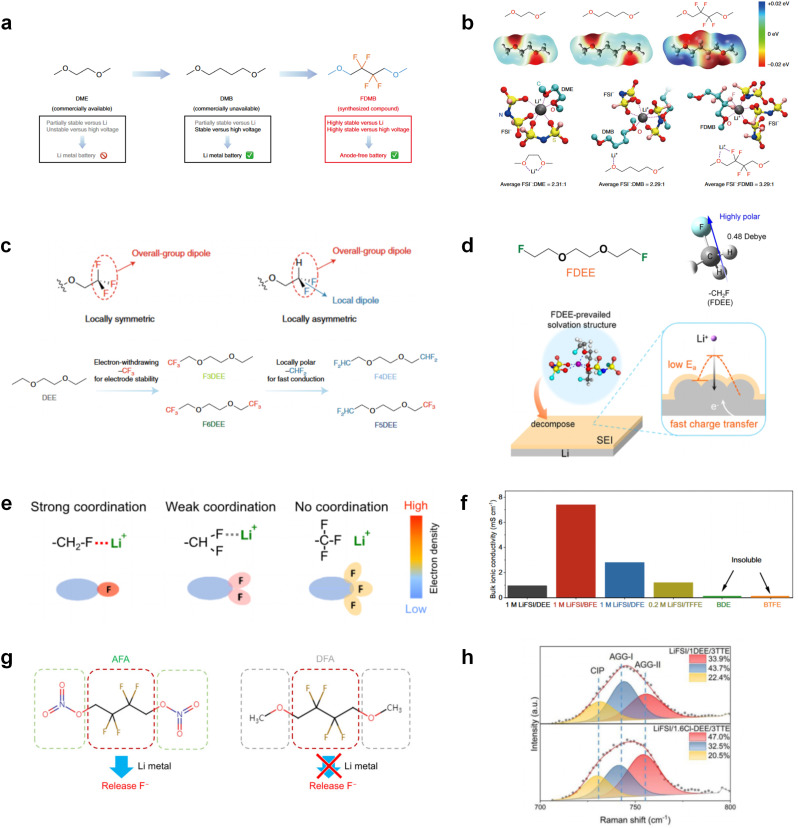
(a) Design scheme and molecular structures of three liquids: DME, DMB and FDMB. (b) ESP comparison of DME, DMB and FDMB and solvation structure of 1 M LiFSI/DME, 1 M LiFSI/DMB and 1 M LiFSI/FDMB. Reproduced with permission. Copyright © 2020, Nature Publishing Group. (c) Logical flow, starting from DEE, for the design of the fluorinated-DEE family. Spatial configuration and dipole direction of –CF_3_ and –CHF_2_. Reproduced with permission. Copyright © 2022, Nature Publishing Group. (d) Illustration of FDEE-prevailed solvation structure forming the SEI with facilitated Li^+^ transport. Reproduced with permission. Copyright © 2023 American Chemical Society. (e) Coordination chemistry of monofluoride, difluoro, and trifluoro groups. (f) Bulk ionic conductivities of various electrolytes at 30 °C, including DEE, BTFE, BDE, TFFE, DFE, and BFE. Reproduced with permission. Copyright © 2023, Nature Publishing Group. (g) Molecular design of AFA with active nitrate ending group and DFA with inert methoxyl on the side of fluorocarbon segment. Reproduced with permission. Copyright © 2022 WILEY-VCH. (h) Raman spectra and the population of AGGs-II (red, 755 cm^−1^), AGGs-I (blue, 744 cm^−1^), and CIPs (yellow, 731 cm^−1^) in the two electrolyte systems. Reproduced with permission. Copyright © 2022 WILEY-VCH.

Although fluorine greatly reduces the electron cloud density of the ether-oxygen, the excessive electron-drawing effect of the –CF_3_ group tends to cause too weak solvation ability of ether molecules. Therefore, the development of other fluorine-containing groups is important for the rational ether molecule regulation. Yu *et al.*^78^ first designed FDMB, in which only the central part of the DMB backbone is replaced with –CF_2_– while the –O– is still linked to CH_3_– and –CH_2_– (Fig. 8a). As a result, the electrolyte using the obtained FDMB molecule possessed unique Li–F binding and high anion/solvent ratio in the solvation sheath, leading to excellent compatibility with Li metal anodes and high-voltage cathodes (Fig. 8b). In order to explore the impact of the fluorinated position on the oxidative stability, Ma *et al.*^121^ shifted the fluorinated segment from the central building block to the terminal group. Surprisingly, the fluorinated position makes a significant difference in the oxidation stability. When the fluorinated segment is located at the terminal, the enhancement of molecular oxidation stability is much more obvious. Furthermore, a family of fluorinated-1,2-diethoxyethanes (F_*x*_DEE) was synthesized and used for electrolyte solvents by Yu *et al.* (Fig. 8c).^79^ The effect of fine modulation of fluorinated groups on the physical and electrochemical properties of ether molecules has been intensively studied, and it was found that partially fluorinated –CHF_2_ with locally polar groups is identified as the optimal group rather than fully fluorinated –CF_3_ (Fig. 8e and f).

Due to the strong electron withdrawing effects of fluorinated substitution groups, the solvents with –CF_2_ or –CF_3_ groups tend to show poor ability in dissolving Li salts and further induce large ion aggregation. This is an issue that cannot be ignored because the interphase composition and structure established by the anion chemistry have shown inadequacy for protecting ultrahigh-voltage cathodes. It was found that the fluorinated group –CHF_2_ is more locally polar than –CF_3_, which enhances the interaction between the fluorinated solvent and Li^+^. Recently, Ruan *et al.*^54^ first reported the design of a monofluoro-ether solvent 1,2-bis(2-fluoroethoxy)ethane (FDEE) with monofluoro (–CH_2_F) group. The highly polar monofluoro –CH_2_F group of FDEE induces a strong interaction with Li^+^ and significantly changes the electrolyte solvation structure (Fig. 8d). Even in highly concentrated electrolyte with rich anion, FDEE can control the reaction on the Li metal anode interphase to induce unique solvent-dominant chemistry. This FDEE solvent-based electrolyte chemistry enables a high Li CE (∼99.4%) and stable Li anode cycling at a high rate (10 mA cm^−2^) due to the high charge transfer and fast Li^+^ conduction of the SEI film, together with greatly improved cycling stability of 4.7 V-class nickel-rich cathodes. Another monofluoride bis(2-fluoroethyl)ether (BFE) shown in Fig. 8e was reported soon as an electrolyte solvent with Li–F and Li–O tridentate coordination chemistries.^122^ The monofluoro substituent (–CH_2_F) in the solvent molecule can improve the electrolyte ionic conductivity (Fig. 8f), which demonstrates good compatibility with high-voltage Li metal batteries in a wide range of temperatures and high charge/discharge rates.

In addition to typical elements like F, the introduction of other functional atoms may also present opportunities for molecular design. As demonstrated in Fig. 8g, Tan *et al.* rationally designed β-chlorine functionality on ether molecular structure and developed a chlorinated solvent 1,2-bis(2-chloroethoxy)-ethyl ether (Cl-DEE).^123^ The electron-drawing effect of chlorine can also decrease the electron cloud density of oxygen like fluorine, thus increasing the oxidation stability of ether-based molecule and forming a weakly solvation electrolyte to enhance the anion activity. This Cl-DEE electrolyte first achieved stable cycling under 4.7 V ultra-high voltage in ether-based electrolytes. At the same time, Cl-DEE solvent decomposition on Li metal anode can bring the LiCl component with high ion transport to the SEI film, which can achieve ∼99.2% Li CE. It is also found that C–Cl bonds are prone to break and release Cl during combustion, which can effectively capture the highly active H·, and the generated HCl further captures OH·. Therefore, Cl-DEE exhibits excellent intrinsic nonflammability. Studies have pointed out that C–F is more difficult to undergo reductive decomposition on Li metal anode, while nitrate is highly reactive. To promote the decomposition of hydrofluoric ethers on Li metal anode to derive fluorinated interphase, Xie *et al.*^124^ proposed a molecular design of activated fluoroalkyl (AFA) in which an active ending group using NO_3_^−^ is attached on each β-site of the fluoroalkyl chain (Fig. 8h). The strong leaving tendency of NO_3_^−^ enables the fast kinetics of fluoride release to render the LiF-rich SEI.

#### 3.1.2 Ester solvent design

Ester solvents containing carbonyl group (C=O) are prone to react with alkali metal anodes resulting from their low LUMO levels. The fluorination of ester-based electrolyte molecules theoretically would further enhance the reactivity of solvents with alkali metals. On the other hand, fluorination can also modulate the solvation structure and form stable fluoride interphases for alkali metal anodes.^31,32,64^ Hence, the reasonable regulation of fluorination degree helps control the electrolyte reactivity with alkali metal interphase and tune the ion conduction of the electrolyte. A typical fluorinated ester is fluoroethylene carbonate (FEC),^125,126^ which is derived from the common EC molecular structure and widely applied as a solvent or additive in graphite- and silicon-based LIBs to construct stable LiF-rich SEI films by molecular decomposition. In the past few years, with the rapid development of alkali-metal batteries, especially LMBs, researchers pay more attention to this effective fluorinated carbonate molecule and also used them to decompose and passivate alkali metal anodes.

**Fig. 9:**
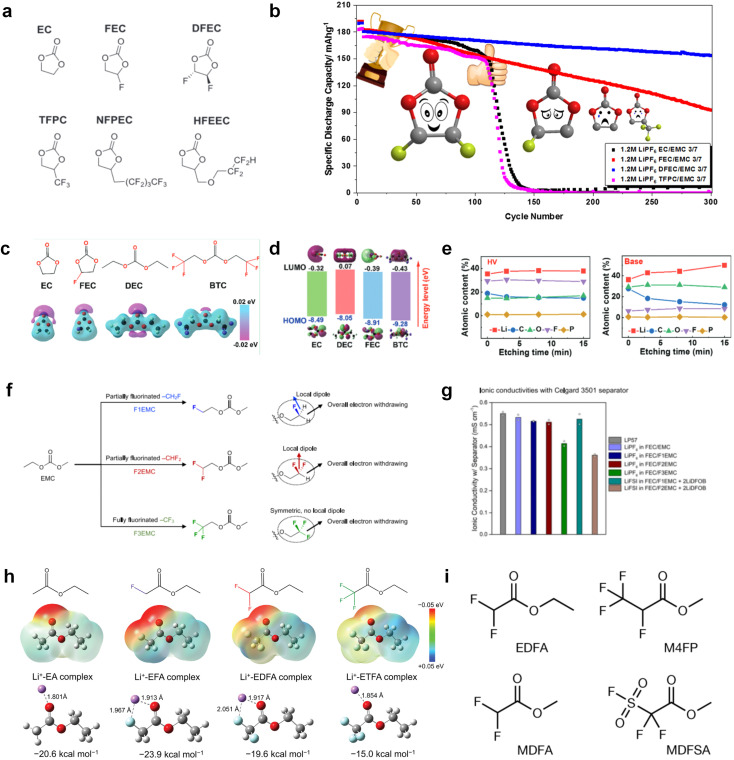
Fluorination of esters. (a) Chemical structures of six cyclic carbonates studied. (b) Cycling performance of Li/NMC622 cells using 1.2 M LiPF6 plus EC/EMC, FEC/EMC, DFEC/EMC, and TFPC/EMC electrolytes (3/7 volume ratios) at room temperature. Reproduced with permission. Copyright 2019, Elsevier Inc. (c) Molecular structures of EC, FEC, DEC, and BTC, and their corresponding calculated charge distributions. (d) Molecular orbital energies of different solvents calculated by DFT. (e) The atomic contents of the SEI in Li‖Li symmetric cells using the base and HV electrolytes derived from in-depth XPS tests at different sputtering times after 10 cycles. (f) Molecular structures of fluorinated-EMCs and schemes to show local and overall dipoles. (g) Ionic conductivity of the electrolytes was measured in coin cells with Celgard 3501 as the separator. Reproduced with permission. Copyright © 2022 IOP Publishing. (h) ESP maps and binding energies of EA and fluorinated-EAs solvents. Reproduced with permission. Copyright © 2023 WILEY-VCH. (i) Chemical structure of the soft solvating solvents. Reproduced with permission. Copyright © 2023, Nature Publishing Group.

Despite the successful application of FEC, there are many structural possibilities for fluorinated cyclic carbonates, and the substituent effect in cyclic carbonates on the electrochemical performance of LMBs is critical to electrolyte optimization. Su *et al.*^127^ designed and synthesized a variety of cyclic carbonates including EC, FEC, DFEC, TFPC, 4-(2,2,3,3,4,4,5,5,5-nonafluoropentyl)-1,3-dioxolan-2-one (NFPEC), and 4((2,2,3,3-tetrafluoropropoxy)methyl)-1,3-dioxolan-2-one (HFEEC) (Fig. 9a). It was found that the substitution of fluorine atoms in cyclic carbonates greatly improves the stability of Li metal anode, while fluoroalkyl and alkoxy substituents are detrimental. Among them, cyclic carbonate *trans*-difluoroethylene carbonate (DFEC) facilitates the formation of a protective SEI with relatively high LiF content (Fig. 9b). In addition, the DFEC-based electrolyte shows better high-voltage compatibility than the FEC-based electrolyte in Li‖NMC622 cells. Furthermore, based on the favorable surface chemistry of FEC on both cathodes and anodes, Aurbach *et al.*^128^ also proposed that the addition of DFEC with a lower LUMO level as a mixed solvent for FEC could significantly improve the cycling performance of practical LMBs. With this hybrid fluorinated electrolyte, the surface film mainly consists of DFEC decomposition during the initial cycling. In the following cycles, FEC acts as a healing agent and maintains the passivation of the Li metal anode. As a result, the consumption of FEC during cycling is significantly reduced and the Li‖NMC622 cells exhibit long cycling. Apart from fluorinated cyclic carbonates, the fluorination of linear carbonates is also effective in improving the compatibility of carbonate electrolytes with alkali metals. Recently, a new fluorinated carbonate solvent bis(2,2,2-trifluoroethyl)carbonate (BTC) was developed by Xiao *et al.* (Fig. 9c)^129^ This designed solvent with a lower LUMO can be preferably reduced on Li metal anode, suppressing Li dendrite growth thanks to the formation of a LiF-rich SEI (Fig. 9d). For the design of fluorinated groups, Yu *et al.*^130^ synthesized a series of carbonate molecules with different fluorinated groups (–CH_2_F, –CHF_2_ and –CF_3_) and compared the reaction differences on graphite anode interphase (Fig. 9f). Although this study focused on graphite anode, this molecular design found that partially fluorinated highly polar –CH_2_F and –CHF_2_ groups may be more beneficial to enhance the ionic conduction of bulk electrolyte and battery performance, which would be helpful to the later investigations of fluorinated carbonate molecules for alkali metal anodes (Fig. 9g).

In addition to the common carbonates, carboxylic esters have gained much attention in recent years due to their low freezing point and therefore are commonly employed in low-temperature batteries. Mo *et al.*^131^ systematically studied a family of fluorinated ethyl acetate molecules to figure out the effect of fluorination on the solvation ability, electrolyte solvation structures and low-temperature properties. It is found that moderately fluorinated ethyl difluoroacetate (EDFA) is more conducive to the reduction of binding energy than low-fluorinated ethyl fluoroacetate (EFA) and more conducive to the dissociation of Li salts than high-fluorinated ETFA (Fig. 9h). Therefore, the EDFA-based electrolyte enables to achieve the graphite anode with a fast-charging capability of up to 6C and excellent cycling performance under low-temperature. Moreover, as shown in Fig. 9i, Xu *et al.*^132^ discovered and investigated the properties of a series of fluorinated esters including EDFA, methyl-2,3,3,3-tetrafluoropropionate (M4FP), methyl difluoroacetate (MDFA) and methyl 2,2,-difluoro-2-fluorosulfonyl acetate (MDFSA). The electrolyte 1 M LiTFSI MDFA/MDFSA-TTE was rationally designed to balance Li^+^-solvent interactions, sufficient salt dissociation and ideal electrochemistry interphases. This novel electrolyte can achieve stable cycling over a wide temperature range (−60 °C to +60 °C) for 4.5 V NMC811‖graphite full cell under high cathode capacity. It is probably because these carboxylates are incompatible with highly active alkali metal anodes even after fluorination, so they are commonly used in graphite anodes. Recently, Mao *et al.*^133^ have screened fluorinated linear carboxylic acid esters (ethyl-3,3,3-trifluoropropionate, tFEP) synergistically with weakly solvating FEC and Li salts (LiBF_4_ and LiDFOB) to induce anion-enrichment interphases. The derived interphases can effectively passivate the Li metal anode and high-voltage cathodes for the stable cycling of high-voltage anode-free LMBs.

Ester solvents show high oxidation stability and can be well matched to high-voltage transition metal oxide cathodes. Nevertheless, their strong reactivity is problematic for conversion cathodes and highly active alkali metal anodes. To stabilize these electrodes, interphase passivation is an effective strategy, such as using some highly reactive salts and increasing salt concentration to involve salt anions in the interfacial reaction. In addition, future molecular design for ester solvents may introduce some effective salt anion groups rather than just fluorination.

#### 3.1.3 Sulfone, sulfonamide and amide solvent design

Similar to carbonate solvents, sulfone- and amide-based solvents are good choices for high-voltage cathodes. Unfortunately, they are also reactive to highly reactive alkali metal anodes, which limits their applications in high-voltage alkali metal batteries.

**Fig. 10:**
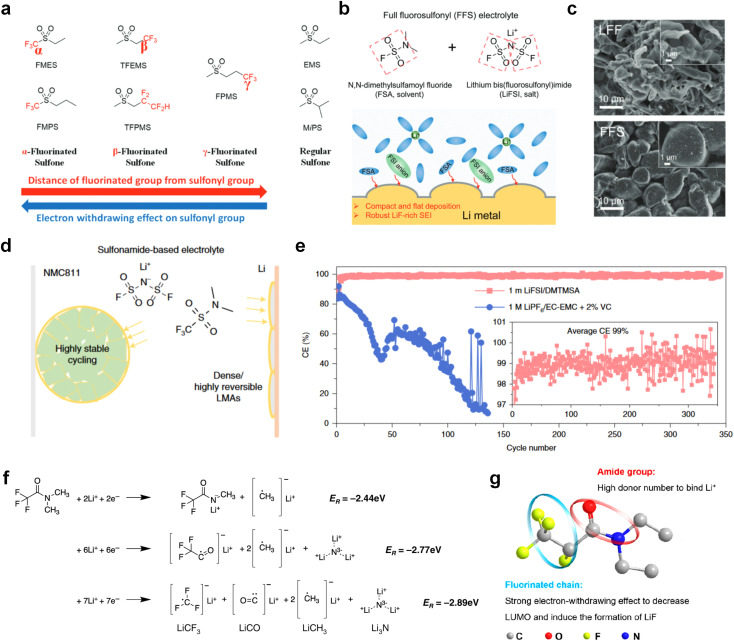
Fluorination of sulfones, sulfonamides and amides. (a) Sulfones with fluorinated group in different position. (b) Design strategy for the FFS electrolyte. The FFS electrolyte is composed of FSA (solvent) and LiFSI (salt) with fluorosulfonyl groups in both components; schematic diagram of Li growth and SEI formation mechanism in the FFS electrolyte. (c) Morphology, surface chemistry and stability analysis of the cycled LMA and SEI layers in different electrolytes. SEM figures of the cycled LMA on Cu substrates in LFF and FFS electrolytes (10 cycles, 0.5 mA cm^−2^, 0.5 mA h cm^−2^) with high resolution SEM images inset. (d) Sulfonamide-based electrolyte can suppress/delay particle cracking and mitigate cathode–electrolyte interfacial reactions, thus enabling a highly stable cathode. This electrolyte also favors the formation of compact uniform Li and suppresses detrimental side reactions, thus enabling a highly reversible LMA. (e) Li metal plating/stripping CEs evaluated by Li‖Cu coin cells at 0.5 mA cm^−2^ and 1 mA h cm^−2^. Inset is the enlarged figure of CEs. Reproduced with permission. Copyright © 2023, Nature Publishing Group. (f) Possible chemical reactions of FDMA on Li-metal surface according to the reaction energy. Reproduced with permission. Copyright © 2020, Nature Publishing Group. (g) Molecular structure of DETFP and its designing principle. Reproduced with permission. Copyright © 2022 American Chemical Society.

The fluorinated design for sulfone molecules may contribute to defluorination of the solvent decomposition to produce LiF-rich SEI film to passivate and stabilize the alkali metal anodes. Su *et al.*^134^ designed and synthesized a new class of fluorinated sulfones as electrolyte solvents (Fig. 10a). α-Fluorinated sulfones and β-fluorinated sulfones were compared for their electrochemical differences. β-Fluorinated sulfones exhibit a significant decrease in reduction potential in contrast to α-fluorinated sulfones, rendering them more stable towards graphite anodes. This fluorination of different sites is a guide to the design of fluorinated sulfone molecules for highly reactive alkali metal anodes. In addition to fluorination, Zhang *et al.*^135^ designed novel sulfone-based molecules by referring to the structure of common LiOTf and PS. Combining the effective passivation for electrodes by salt decomposition and the strong oxidative stability of sulfone groups, the electrolyte consisting of 1.9 M LiFSI, 2,2,2- trifluoroethyl trifluoromethanesulfonate and 2,2,2-trifluoroethyl trifluoromethanesulfonate realized long cycling stability for 4.55 V graphite‖LCO and 4.6 V graphite‖NMC811 batteries.

Moreover, the FSI^−^ anion is often applied to passivate alkali metal anodes due to its high reduction stability and the high activity of S–F, which can be easily defluorinated.^71^ Xue *et al.* were inspired by the fluorosulfonyl imide group from the well-known salt LiFSI and designed an organic solvent dimethylsulfamoyl fluoride (FSO_2_NC_2_H_6_), a fluorosulfonamide (FSA) with two methyl substituents (Fig. 10b).^136^ This molecule combined with LiFSI to form a 1 m “full fluorosulfonyl” (FFS) electrolyte to enable a highly reversible LMA with an excellent initial CE of ∼91%, rapidly approaching 99% within only 10 cycles (Fig. 10c). Furthermore, the S–F of the FSO_2_NC_2_H_6_ molecule was designed into S-CF_3_ to obtain *N*,*N*-dimethyltrifluoromethane-sulfonamide (DMTMSA) (Fig. 10d).^137^ A regular concentration of 1 m of LiFSI with DMTMSA enables highly reversible LMAs of average 99% CE over 300 cycles by favoring compact Li metal deposition morphologies and minimizing pulverization (Fig. 10e). Both the sulfonamide-based organic solvents achieve compatibility with high oxidation stability and high Li reversibility.

Amides, like sulfones, show excellent oxidation resistance, but the stability of alkali metal anodes still remains a challenge. Similar to the sulfone, fluorination strategies are applicable to amide-based molecular design. Wang *et al.*^138^ proposed an amide-based solvent 2,2,2-trifluoro-*N*,*N*-dimethylacetamide (FDMA) in 1 M lithium bis(trifluoromethanesulfonyl)imide mixed with FEC to decompose and construct F-containing and inorganic-rich interphases, which exhibits high Li reversible CE and cycling stability of high-voltage LMBs. In addition, Zhou *et al.*^139^ designed a fluorinated amide molecule named *N*,*N*-diethyl-2,3,3,3-tetrafluor-opropionamide (DETFP) with an amide group to bind Li^+^ and a fluorine chain to induce the formation of LiF. This molecule can be used as an additive to regulate interfacial reactions in carbonate electrolytes to achieve excellent performance of Li‖LiFePO_4_ at 5C.

#### 3.1.4 Phosphate solvent design

Organic phosphates are known for their non-flammability, and the phosphorus atoms in them act as trapping agents for the radicals that initiate the chain reaction and lead to combustion.^140–142^ However, these organic phosphates are unable to form a stable SEI film. Functionalized atomic modification like the fluorine of phosphate molecules can effectively solve the interfacial film instability issue. Zheng *et al.*^143^ designed and synthesized a five-membered fluorinated cyclic phosphate solvent by imitating the chemical structure of EC, incorporating the non-flammability of the phosphate group and introducing the –CF_3_ group. Electrolytes based on such molecules can passivate the graphite anode to enable reversible lithiation and delithiation. This successful design provides an effective strategy for phosphate molecules to be compatible with alkali metal anodes. The functionalized structural design for phosphate-based molecules has not been much studied so far probably due to the interfacial instability towards alkali metal anodes. In the future, it would be interesting to develop phosphate molecules not only limited to fluorination strategies.

#### 3.1.5 Co-solvents or diluents design

In common dilute concentration (1 M) electrolytes, the solvation shell contains a lot of solvents, while a significant amount of free solvents is also present in the electrolyte. As a result, the solvent is susceptible to reductive decomposition on the highly reactive alkali metal anodes. Thus, the organic-based SEI film cannot effectively passivate the electrode and the further electrolyte decomposition. Increasing the salt/solvent ratio to obtain highly concentrated electrolyte (HCE, > 3 M) can significantly induce CIPs and AGGs clusters in the solvation structure while weakening the coordination between the solvent and the cation.^71,95,144^ The HCE electrolytes can reduce the decomposition of the solvents and promote more inorganic species in the derived SEI film, which significantly enhances the antioxidant ability of the electrolytes and helps passivate the highly reactive alkali metal anodes. However, the viscosity of the HCE electrolyte increases significantly with enhancing salt concentration, making it difficult to wet the electrode material and separator.^145^

**Fig. 11:**
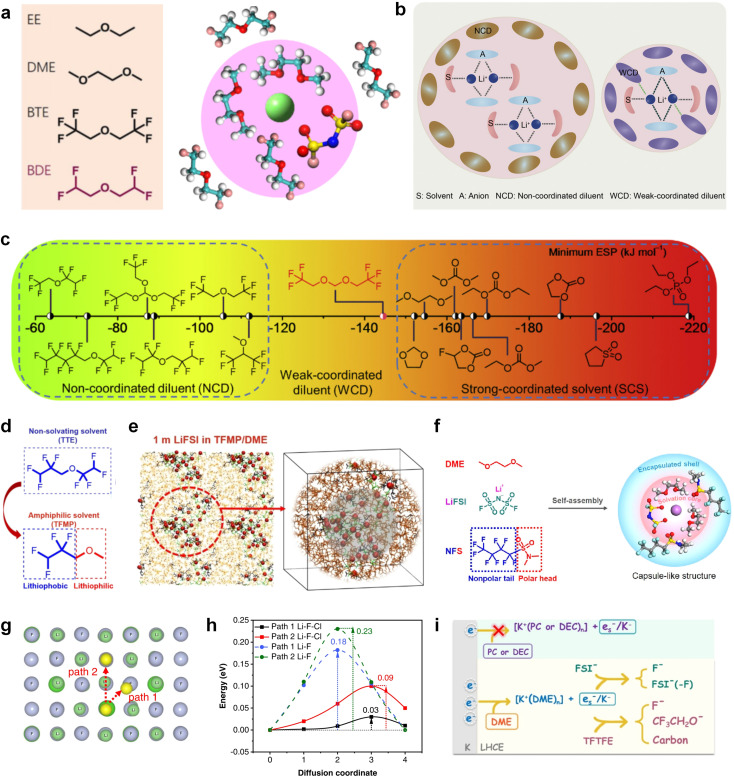
Diluent and cosolvent designs. (a) Scheme of NCD and WCD. Reproduced with permission. Copyright 2022, Elsevier Inc. (b) The proposed unique solvation structure of the BDE/DME electrolyte. Reproduced with permission. Reproduced with permission. Copyright 2022, Elsevier Inc. (c) Calculated minimum ESP of the reported diluents and solvents. Reproduced with permission. Copyright 2022, Elsevier Inc. (d) Amphiphilic solvent design strategy for the LMB electrolyte. (e) MD simulation snapshots with 2 × 2 boxes of 1 M LiFSI-TFMP/DME electrolytes. Reproduced with permission. Copyright © 2023 WILEY-VCH. (f) Molecular design principle of the bipolar NFS molecule and the corresponding capsule-like solvation structure. Reproduced with permission. Copyright © 2023 American Chemical Society. (g) Schematic illustration of Li^+^ diffusion path. (h) Variation of NEB energies with path 1 and path 2 along LiF or LiF_1−*x*_Cl_*x*_ grain boundaries. Reproduced with permission. Copyright © 2022, Nature Publishing Group. (i) The distinct decomposition pathways in ether or ester solvents due to different capabilities for forming K^−^/e_s_^−^. Reproduced with permission. Copyright © 2022 WILEY-VCH.

Furthermore, a state-of-the-art LHCE was developed by adding non-polar organic molecules such as HFE to the HCEs, which maintains the solvation structure of HCEs while achieving a low viscosity for the electrolyte.^74,75,146–148^ This emerging electrolyte has been successfully employed in many battery systems including alkali metal batteries. Nevertheless, the transport of Li^+^ in LHCEs is limited due to the difficulty of such non-polar diluent molecules to participate in Li^+^ coordination, which leads to poor rate performance.^54^ BTFE, a common HFE diluent with a high F/O ratio, is hard for oxygen to be involved in the coordination of the solvation structure. Zhang *et al.*^149^ designed a partially fluorinated BTFE-based molecule named bis(2,2-difluoroethyl)ether (BDE, Fig. 11a) with the –CHF_2_ group. The BDE molecule serves as a diluent and co-solvent to improve the electrolyte ionic conductivity by interacting with Li^+^. In the meantime, BDE promotes the formation of uniform LiF-rich SEI to suppress dendrite growth by regulating the solvation shell structure, resulting in a high Li CE of 99.6%. Similarly, Zhao *et al.*^150^ developed a weak-coordinated diluent (WCD, Fig. 11b and c) by designing a new HFE molecule bis(2,2,2-trifluoroethoxy)methane (BTFM), which enhances the interaction between the diluent and Li^+^ by adding an oxygen to the BTFE molecule. The LHCE using BTFM can enable the formation of an inorganic-rich SEI with ultra-high Li_2_O content, demonstrating stable Li reversibility and excellent rate performance for LMBs. Additionally, based on the structure of BTFE, reducing the fluorine content also strengthens the combination between oxygen and Li^+^, promoting the diluent into the solvation shell. It is noted that this WSD design rationalizes the ESP of different molecules to represent their polarity to reflect the binding ability to cation, which can help to classify the solvent distribution in the inner or outer solvation layer. This quantitative representation provides a reference for the future precise design of molecular structures with suitable polarity.

Another effective strategy to improve the coordination of the diluent and Li^+^ is to introduce or expose polar groups in the molecular structure. Zhang *et al.*^151^ synthesized a bipolar molecule with an ion dissociative polar head and a perfluorinated nonpolar tail (TFMP, Fig. 11d and e). The bipolar solvent promotes the formation of capsule-like solvation sheaths by weak coordination and enclosing the polar heads inside the primary solvation shell, which helps reduce the detrimental decomposition of solvents. Shi *et al.*^152^ proposed to remove the F-containing group on one side of the TTE diluent to expose the O site, allowing the diluent to coordinate with Li^+^ (Fig. 11f). The obtained amphiphilic diluent molecule 1,1,2,2-tetrafluoro-3-methoxypropane enables LHCE to not only improve the Li^+^ transport and exhibit lower desolvation energy to achieve facile desolvation but also promotes the formation of a robust and conductive inorganic SEI. This electrolyte demonstrates a high Li efficiency of 99.6% at room temperature and significantly improved cycling stability at −40 °C for more than 100 cycles.

In addition to HFEs with high F/H ratios, alkanes have recently been investigated for use as diluents in LHCEs. Wu *et al.*^153^ screened the possible diluents and found that 2H,3H-decafluoropentane (HFC) satisfies the principle of relatively weak but sufficient interactions with solvation shell, which makes it suitable as the diluent. This fluorinated alkane can strengthen the Li^+^ coordination and offer the LHCE high antioxidant ability for 6 V through the floating test of the leakage current. Thus, the LHCE with HFC achieved practical high-voltage LMBs to realize high capacity retention with a full-cell CE of 99.91%. Additionally, Zhang *et al.*^154^ proposed the use of a new chlorinated alkane 1,2-dichloroethane (DCE) as a diluent for LHCEs, and it was found that the co-decomposition of high concentration FSI^−^ and chlorinated diluents can derive dual-halide (LiF_1−*x*_Cl_*x*_) SEI, where Cl doping can endow the LiF_1−*x*_Cl_*x*_ phase with fast Li^+^ conductivity (Fig. 11g and h). This electrolyte design provides an important reference for the construction of high-quality interphases for both cathodes and alkali metal anodes.

It is worth noting that even though the advanced ether LHCEs can greatly enhance the electrochemical performance of AMBs, it is difficult for them to have compatibility with higher activity sodium and potassium metals, mainly because the strong complexation between ether solvents and alkali metal cations can promote the dissolution of alkali metals. At the same time, alkali metal anions (M^−^) and solvation electrons (e_s_^−^) are further generated, which leads to the failure of alkali metal anodes. Recently, Chen *et al.*^155^ systematically explored the compatibility between LHCEs and alkali metal anodes and found that the alkali metals (Li, Na, K) can be corroded by electrolytes after long cycling. Finally, the failure mechanism of alkali metal anodes in ether-based LHCE mediated by phase transfer is proposed (Fig. 11i), which provides a new perspective on the interfacial reactions of LHCEs and alkali metal anodes.

#### 3.1.6 Salt design

As one of the most important components in the electrolyte, salts play a crucial role in passivating highly active cathodes and alkali metal anodes. For example, for LMBs, the reaction between the electrolyte and Li metal is inevitable and the key factor is whether an effective passivation layer can be formed to isolate the Li metal from further reactions. Common Li salts, such as LiPF_6_, LiBF_4_, LiClO_4_, LiBOB, LiDFOB, LiFSI, and LiTFSI, are commonly used in electrolytes. However, the salts with ClO_4_^−^ and BF_4_^−^ anions are seldom investigated due to their low solubility and inferior ionic conductivity in common organic solvents. Though the PF_6_^−^ anion could passivate the Al collector, the PF_6_^−^ anion-based electrolytes usually suffer from poor cycle stability and low Li CE, stemming from their insufficient passivation ability on the anode surface and inferior oxidation resistant properties. The fully reduced or partially reduced products of these salts are often found in the SEI, such as LiF and Li_*x*_PF_*y*_O_*z*_ from the reduction of LiPF_6_, LiF and Li_*x*_BF_*y*_O_*z*_ from the reduction of LiDFOB, and so on.

The reduction decomposition mechanisms of these salts are listed in Table 1.^156^ It shows that LiFSI exhibits a simpler decomposition path relative to other common salts and is more likely to produce LiF and sulfide-containing compounds, which are considered to be effective interfacial components. Additionally, most electrolytes using LHCE structures employ LiFSI as the primary salt, benefiting not only from its strong solubility but also from its ability to form a stable passivation layer. It should be noted that LiTFSI has stronger solubility in ether or carbonate-based electrolytes, but the C–F bond in LiTFSI is less prone to break compared to the S–F bond in LiFSI. As a result, LiTFSI has a weaker passivation capability and cannot effectively form an inorganic-rich passivation layer at the interface compared with LiFSI. Instead, organic compounds containing the –CF_3_ group are formed. Unfortunately, both dilute FSI^−^ and TFSI^−^ anion-based electrolytes will cause corrosion on the Al collector under high voltage. Therefore, there is an urgent need to synthesize novel salts for high performance alkali metal batteries.

**Table 1:** The reduction decomposition mechanisms of Li salts

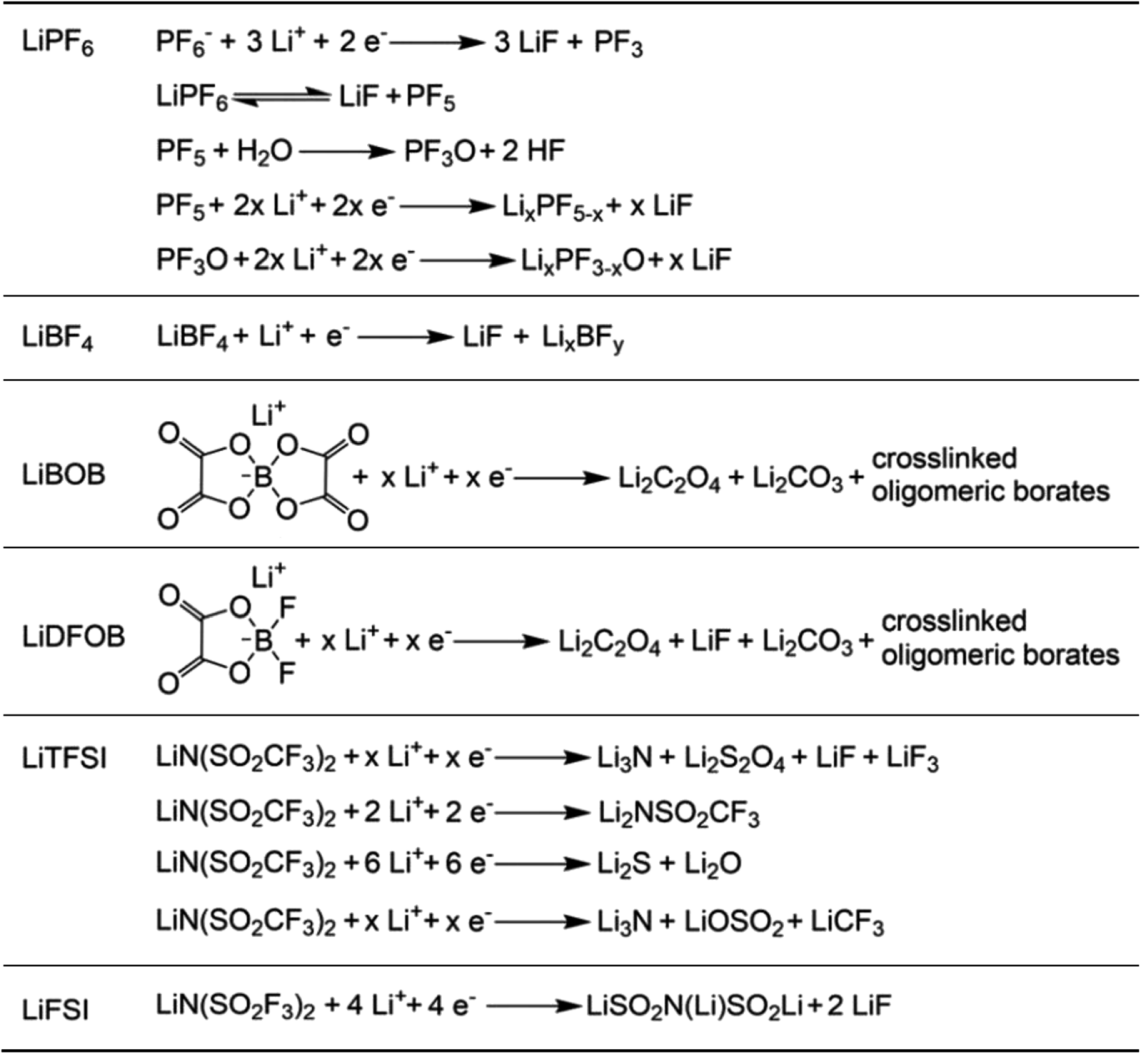

**Fig. 12:**
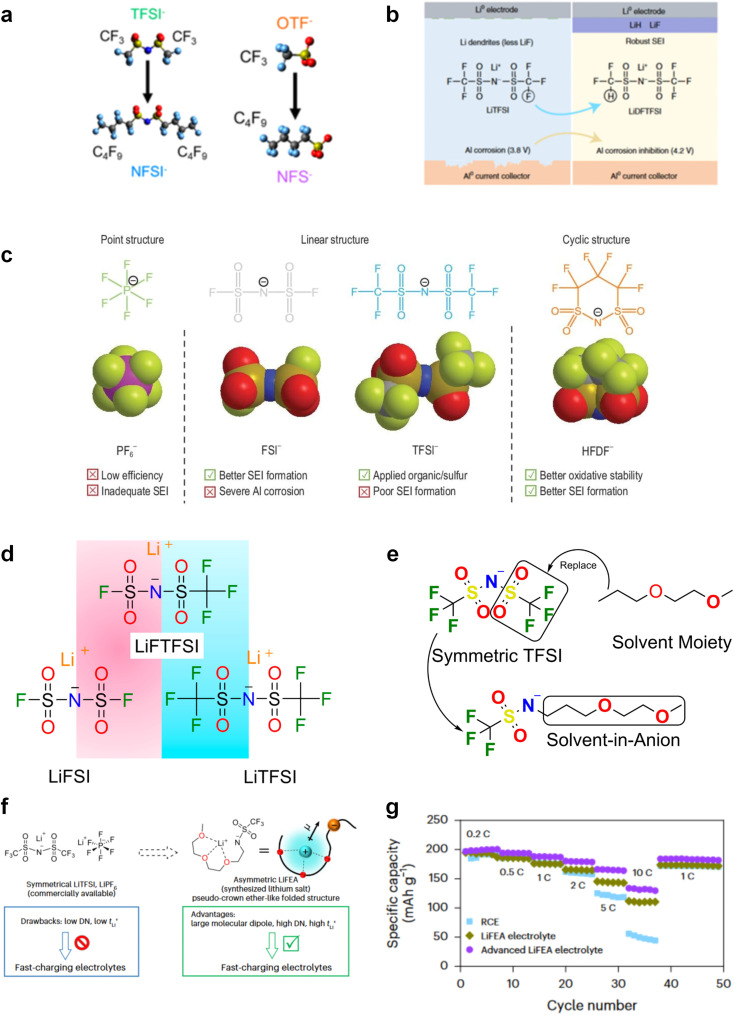
(a) Molecular visualization of fluorocarbon chain length modification in bis(C_*x*_F_*y*_ sulfonyl) imide and C_*x*_F_*y*_ sulfonate moieties. Reproduced with permission. Copyright © 2022 American Chemical Society. (b) The design concept of LiDFtFSI and its unique properties in comparison with LiTFSI in 4 V-class LMBs. Reproduced with permission. Copyright © 2022, Nature Publishing Group. (c) Design scheme and molecular structure of the anions HFDF^−^. (d) Concept of solvent-in-anion design. Reproduced with permission. Copyright © 2020 American Chemical Society. (e) Comparison of the chemical structures for the LiFTFSI, LiFSI, and LiTFSI salts. Reproduced with permission. Copyright © 2018 American Chemical Society. (f) Design principle of LiFEA. (g) Rate performance with RCE, LiFEA electrolyte and advanced LiFEA electrolyte. Reproduced with permission. Copyright © 2023, Nature Publishing Group.

As mentioned above, salts can significantly affect the physical properties of the electrolyte and the performance of the alkali metal batteries thanks to the behavior of anions and cations at the interphase, especially the anion. TFSI^−^ and OTF^−^ are common salt anions often paired with ether solvents, but ether electrolytes utilizing these salts are known to be incapable of operating above 4 V. In addition to the instability of solvent, the corrosion of the current collector Al at a high voltage (∼3.8 V) is also a serious problem. By substituting the terminal –CF_3_ groups with –C_4_F_9_ chains, Holoubek *et al.*^157^ (Fig. 12a) present a possible route forward for improving the oxidation stability of these electrolytes, which enabled the reversible cycling of LiNi_0.8_Mn_0.1_Co_0.1_O_2_ at a cutoff of 4.4 V in electrolytes consisting of only 1 M salt and DME. This enhanced oxidation stability was driven by a passivated interphase composed largely of perfluoroalkane species, which is consistent with the previous results that organic fluorine species are beneficial for high voltage cathode cycling due to the solvophobic nature of perfluoroalkyl groups. Hence, Li salts with increased fluoroalkane chains or fluoroalkayl phosphate may be promising directions. The replacement of the –CF_3_ moiety with longer perfluorinated alkyl chains could effectively widen the electrochemical stability of the Al^0^ current collector but at the high expense of lowering the ionic conductivity. Similarly, Qiao *et al.*^158^ reported an additive-free, carbonate-based electrolyte using a non-corrosive sulfonimide salt, LiDFTFSI (Li[N(SO_2_CF_2_H)(SO_2_CF_3_)] (Fig. 12b) to remarkably improve the performance of 4 V-class rechargeable LMBs. Intuitively, one may anticipate that LiDFTFSI would be less resistant to oxidation than LiTFSI due to the lower electronegativity of H atoms compared with F atoms (for example, 2.2 (H) *versus* 3.98 (F) on the Pauling scale), and that its electrolyte may still be corrosive towards the Al^0^ current collector due to the structural similarity of the two salts, which has been witnessed in electrolytes with other sulfonimide salts. Surprisingly, the LiDFTFSI-based electrolyte is anodically stable for 4 V-class cathodes and does not corrode the Al^0^ current collector at a high potential of at least 4.2 V *versus* Li/Li^+^. It was attributed to the formation of a thin and robust protective layer with a balanced proportion of AlF_3_ and LiF resulting from the decomposition of the DFTFSI^−^ anion. In addition, the stability of salts to the alkali metal anodes also affects the performance of full cells. Nevertheless, it is often difficult to be compatible with both the cathode and anode. As shown in Fig. 12c, Hu *et al.*^159^ found that common anions fail to simultaneously inhibit the dead K formation of potassium metal anode and the aluminum collector corrosion of high-voltage cathode and proposed a cyclic hexafluoropropane-1,3-disulfonimide anion (HFDF^−^). This novel anion can effectively passivate cathode and anode interphases and thus enhance the cycling stability for high-voltage AMBs. Interestingly, for Li–S batteries, LiTFSI and LiFSI are stabilized for sulfur cathode and Li metal anode, respectively. This is because the S–F of LiFSI may react with polysulfides yet passivates the Li metal more effectively compared to LiTFSI. Based on this, Eshetu *et al.*^160^ introduced both S–F and –CF_3_ into the anion to balance the stability of the anode and cathode sides (Fig. 12d). The designed salt was combined with PEO to form a solid electrolyte, realizing the high capacity and excellent rate performance of Li–S batteries. This composite structural molecular design is also shown in other works. For example, Luck *et al.*^161^ proposed to introduce ether structures into the sulfonamide anion to obtain a series of novel salts that exhibit high solubility in ether-based solvents. Such salts can be used in potassium–oxygen batteries to effectively reduce the overpotential and improve the electrochemical stability of cathode (Fig. 12e). Recently, Xia *et al.*^162^ also combined the ether chain with the sulfonamide anion to develop an asymmetric salt, lithium 1,1,1-trifluoro-*N*-[2-[2-(2-methoxyethoxy)ethoxy)]ethyl] methanesulfonamide (LiFEA), which shows a pseudo-crown ether-like, folded molecular geometry (Fig. 12f). This salt enables carbonate electrolyte to possess a large apparent donor number and Li^+^ transference number and also drives a self-cleaning mechanism for SEI, which enhances the cycling performance of practical LMBs under high discharge current density.

**Fig. 13:**
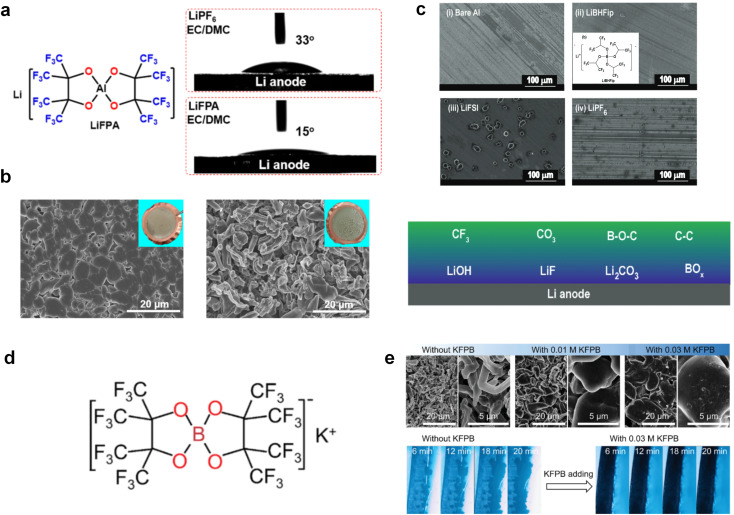
Salt designs. (a) Contact angle of 1 M LiPF_6_-EC/DMC and 1 M LiFPA-EC/DMC to Li anode. (b) Morphology of deposited Li on Cu foil using 1 M LiFPA-EC/DMC and 1 M LiPF_6_-EC/DMC at a current density of 0.5 mA cm^−2^ for 16 h. Insets are optical images of deposited Li on Cu foil. Reproduced with permission. Copyright © 2022 American Chemical Society. (c) Schematic representation of the species distribution in the Li electrode SEI. Reproduced with permission. Copyright © 2021 WILEY-VCH. (d) Molecular structure of KFPB. (e) Typical SEM images of deposited Li in BE electrolyte without and with 0.01/0.03 m KFPB (0.5 mA cm^−2^ and 4 mA h cm^−2^). *In situ* optical microscopy observations of Li plating at a current density of 5 mA cm^−2^ in BE and BE with 0.03 m KFPB. Reproduced with permission. Copyright © 2023 WILEY-VCH.

It can be noted that among the anions, F, O, S, and P are common elements that contribute to the interphases. In recent years, the application of other elements in salt anions has also been studied. Aluminum (Al)-containing compounds (such as Al_2_O_3_ and AlF_3_) coatings can improve the interfacial structure stability of cathode materials. But coating Al-containing compounds always requires additional high-cost calcination processes. Li *et al.*^163^ synthesized a highly fluorinated (8-CF_3_) aluminum (Al)-centered Li salt of lithium perfluoropinacolatoaluminate (LiFPA, Fig. 13a). This LiFPA salt exhibits good interfacial compatibility with Li metal anode by reducing electrolyte surface tension, thus enhancing electrolyte wettability and ultimately suppressing Li dendrite growth (Fig. 13b). By means of electrolyte decomposition, at the cathode side, the LiFPA salt facilitates the *in situ* formation of a passivating CEI layer enriched with Al_2_O_3_, AlF_3_, and LiAlF_4_, and enables the practical Li metal batteries (LMBs) with good cycling stability at a high voltage (4.3 V). Additionally, B-based salts also show high potential for adoption, such as the common LiBF_4_ and LiDFOB. For instance, Roy *et al.*^164^ synthesized and investigated lithium 1,1,1,3,3,3-(tetrakis)hexafluoroisopropoxy borate (LiBHfip). It is found that salt decomposition can construct the SEI rich in inorganic LiF together with borate and CF_3_-rich organic species, which is good for the suppression of Li dendrites (Fig. 13c). In addition, the design of salt additives is also a solution to improve the electrolyte compatibility with alkali metal anodes. As shown in Fig. 13d, another B-based salt potassium perfluoropinacolatoborate (KFPB) was designed as an additive by Zhang *et al.*^165^ FPB^−^ anion possesses a strong adsorption ability on Li anode, which preferentially adsorbs and decomposes on the Li anode surface to construct a conductive and robust SEI. Meanwhile, Li^+^-FPB-and K^+^-PF_6_-ion-pairs with low LUMO energy were promoted to form the inorganic SEI. Thus, the carbonate electrolyte adding KFPB additive exhibits the effective suppression of Li dendrites and excellent Li plating/stripping stability (Fig. 13e).

Salt, as one of the important components of the electrolyte, does more than providing anions and cations in the bulk electrolyte. The anions tend to be involved in the interfacial reactions, and their structure and components can determine the properties of the interphase. Therefore, the rational design of the anion structure can not only effectively change the physicochemical properties of the electrolyte but also regulate the interfacial electrochemical reactions. Currently, the common anion design ideas are mainly to passivate the electrode by introducing F elements or utilizing highly reactive anion structures. In the future, it may be a new and effective strategy to design salt compositely from the solvent structures.

#### 3.1.7 Additive design

**Fig. 14:**
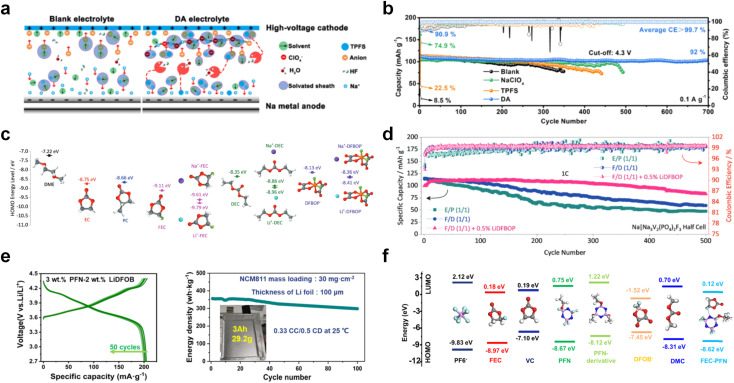
Additive designs. (a) The dynamic high-voltage resistance and self-purifying mechanism. (b) Cycling performances of Na‖NVPF batteries with a cut-off voltage of 4.3 V. Reproduced with permission. Copyright © 2022 WILEY-VCH. (c) HOMO energy levels of DME, EC, PC, FEC, Na^+^-FEC, Li^+^-FEC, DEC, Na^+^-DEC, Li^+^-DEC, DFBOP^−^, Na^+^-DFBOP^−^, and Li^+^-DFBOP^−^ obtained by DFT calculations. (d) Cycling performance of Na‖Na_3_V_2_(PO_4_)_2_F_3_ half cells operated in different electrolytes at 1C. (e) Charge/discharge curves of 35 μm Li‖NMC811 (6 mA h cm^−2^) at 0.33C/0.5C in the optimal electrolyte. Cycling performance of 3 Ah pouch cell in the optimized electrolyte with electrolyte/capacity of 2.5 g A h^−1^. (f) Frontier molecular orbitals of electrolyte components and additives. Reproduced with permission. Copyright 2022, Elsevier Inc.

The application of additives in electrolytes is a simple and effective method to improve the performance of alkali metal batteries.^166,167^ For example, Fig. 14a and b show that Wu *et al.*^168^ proposed an anion/cation solvation strategy for realizing 4.7 V-resistant SMBs electrolyte with NaClO_4_ and trimethoxy-(pentafluorophenyl)silane (TPFS) as dual additives (DA). ClO_4_^−^ can rapidly transfer to the cathode surface and strongly coordinate with Na^+^ to form stable polymer-like chains with solvents. Meanwhile, TPFS can preferentially enter into the PF_6_^−^ anion solvation sheath for reducing PF_6_^−^ solvent interaction and effectively scavenge adverse electrolyte species for protecting electrode electrolyte interphases. Thus, such an electrolyte elevates the oxidation stability of carbonate electrolytes from 3.77 to 4.75 V. To meet the catalytic nature of aggressive high-voltage chemistries, Chen *et al.*^169^ fabricated a tailored carbonate-based electrolyte involving lithium difluorobis(oxalato) phosphate (LiDFBOP, Fig. 14c and d) as a multifunctional additive, where DFBOP^−^ anions can generate stable interphases on the cathode. Meanwhile, Li^+^-ions can take part in the solvation structure to regulate the electrolyte stability. Although FEC is widely recognized as an effective cosolvent or additive coupling with commercial carbonate-based electrolyte to enable stable high-voltage LMBs, however, it reacts with the decomposition product (PF_5_) of the LiPF_6_ salt in the presence of a trace amount of water, producing corrosive species such as HF and thus deteriorating the stability of the LMBs. Hence, Zhang *et al.*^170^ employed a nonnucleophilic organic base, ethoxy(pentafluoro)cyclotriphosphazene (PFN), as a multifunctional additive to simultaneously enhance the chemical stability and safety of the FEC-LiPF_6_ carbonate-based electrolytes (Fig. 14e and f). The electron-donating –P=N– group of PFN enables it to catch the Lewis acid PF_5_ and HF in the electrolyte, while the steric hindrance from the resembling benzene structure of PFN prevents it from attacking the FEC. Furthermore, the PFN additive also improves the safety and processability of the battery since it has a superior flame retardancy and good wettability with the separator. As a result, adding 3 wt% of PFN gives an FEC-containing carbonate electrolyte that is self-extinguishing, which significantly improves the stability of high-voltage Li‖NMC811 batteries, especially when it couples with lithium difluoro(oxalato)borate (LiDFOB) as a film-forming additive.

### 3.2 For alkali metal batteries with conversion-type cathode

#### 3.2.1 Metal–sulfur batteries

**Fig. 15:**
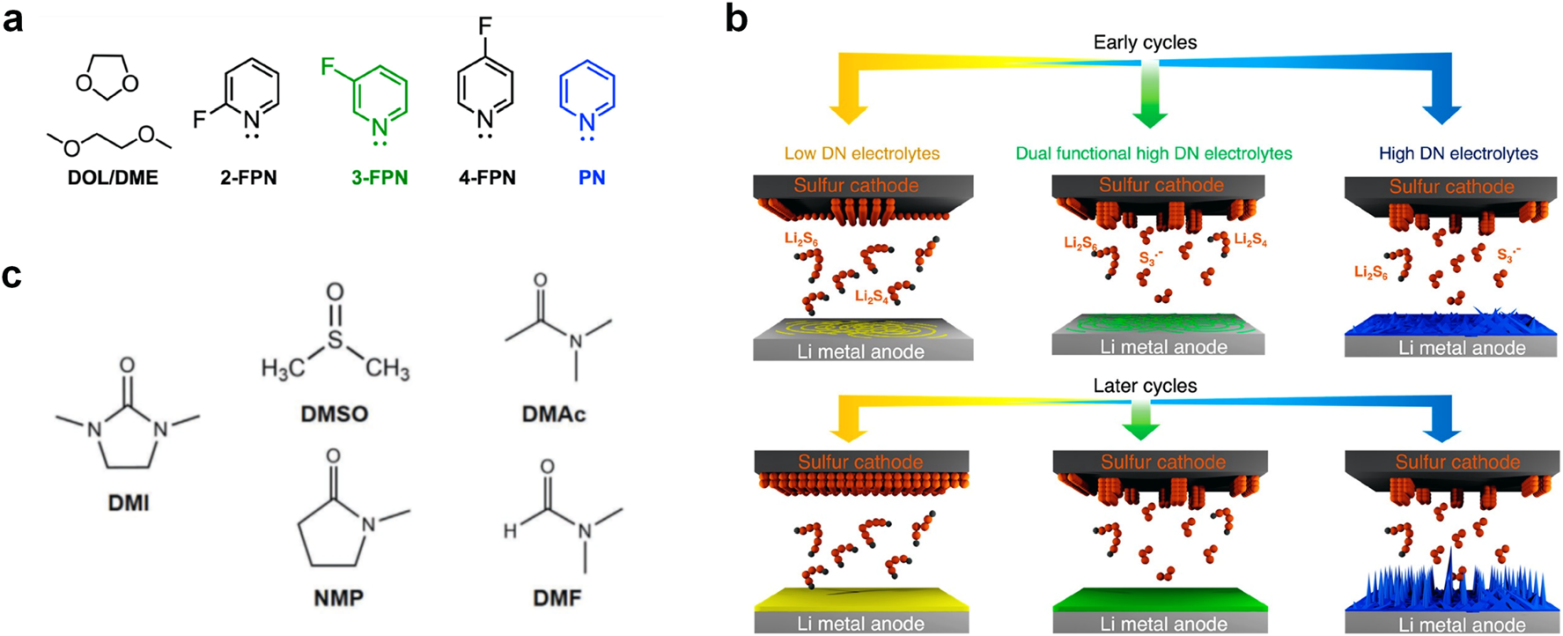
(a) Chemical structures of 1,3-dioxolane, dimethoxyethane (DOL/DME), pyridine (PN) and its –F substituted derivatives, namely, 2-FPN, 3-FPN, and 4-FPN. (b) Schematic illustration of operating mechanisms in Li–S cells when the electrolytes with different donor abilities and functions are used: low DN electrolyte, dual functional high donor electrolyte, and high DN electrolyte. Copyright © 2022 American Chemical Society. (c) Molecular structures of high donor electrolyte solvents. Reproduced with permission. Copyright © 2020 WILEY-VCH.

As a cathode material with ultra-high capacity, sulfur is used in metal-based batteries to build high energy density battery systems and thus receives a lot of attention from researchers in recent years. In this conversion-type cathode material, the interfacial reaction kinetics between the solid and liquid phases is the key to improving the active utilization of the sulfur. Electrolyte engineering, especially designing new solvent molecules, can improve solid–liquid interfacial reactions. Elabd *et al.*^171^ introduced a dual functional high donor solvent, 3-fluoropyridine (3-FPN), to achieve high polysulfide solubility and compatibility with Li metal (Fig. 15a and b). The use of strong polar molecules accelerates the kinetics of the cathode interphase reaction, effectively enhancing the capacity release and cycling stability of Li–sulfur batteries (LSBs) under lean electrolyte condition. Similarly, as shown in Fig. 15c, 1,3-dimethyl-2-imidazolidinone (DMI) was reported as a new high donor electrolyte for LSBs by Baek *et al.*^172^ The high solubility of polysulfides in DMI allows it to activate a new reaction route, which engages the sulfur radical (
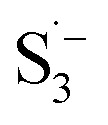
), thus enabling the efficient utilization of sulfur.

#### 3.2.2 Metal–air batteries

**Fig. 16:**
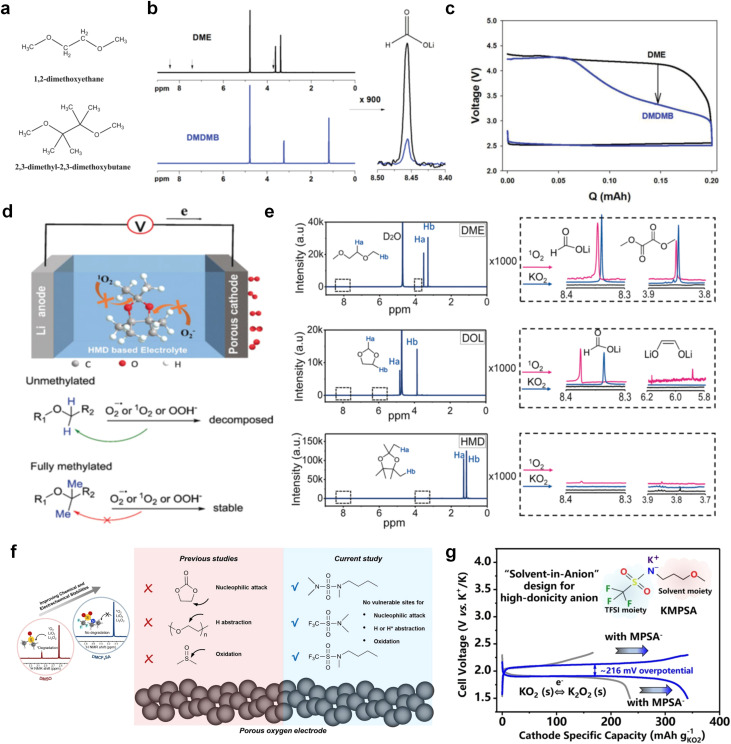
(a) The structures of DME and 2,3-dimethyl-2,3-dimethyoxybutane (DMDMB). (b) The ^1^H-NMR spectra of side-products deposited on cathodes after 1st discharge in [(DME)2Li]TFSI and [(DMDMB)2Li]TFSI. (c) The first galvanostatic discharge–charge cycle for the [(DMDMB)2Li]TFSI and [(DME)2Li]TFSI electrolytes at current densities of 50 μA cm^−2^. Reproduced with permission. Copyright © 2015 WILEY-VCH. (d) Comparison of the reaction activity of unmethylated and fully methylated cyclic ether. (e) ^1^H NMR spectra of DME, DOL and HMD before (black) and after reaction with ^1^O_2_ (pink) or KO_2_. Reproduced with permission. Copyright © 2018 WILEY-VCH. (f) Dominant degradation mechanisms of carbonate-, ether-, and sulfoxide-based electrolytes and the molecular design of stable sulfamide- and sulfonamide-based solvents BTMSA (top), DMCF3SA (middle), and BMCF3SA (bottom) for aprotic Li–O_2_ batteries. Reproduced with permission. Copyright 2019, Elsevier Inc. (g) Following the concept of “Solvent-in-Anion” design, the asymmetric anion (MPSA^−^) with high donor ability was synthesized and validated to boost the reversibility of potassium superoxide/peroxide redox without any electrocatalysts. Reproduced with permission. Copyright © 2023 WILEY-VCH.

Metal-based batteries using air as the cathode possessing a high theoretical energy density have attracted much attention in recent years.^29,73,173–175^ However, the electrolyte instability tends to react with the reaction intermediate of the cathode, thus increasing the overpotential and reducing the cycling stability of the battery. The rational regulation of the electrolyte, especially the design of new stable solvent molecules, is expected to solve the problem of strong reactivity between the electrolyte and cathode. As shown in Fig. 16a, Adams *et al.*^176^ synthesized a lithium ether-derived chelating ionic liquid 2,3-dimethyl-2,3-dimethoxybutane (DMDMB) as an electrolyte solvent for Li–O_2_ batteries (LOBs). The ether framework is more intrinsically stable to superoxide-initiated hydrogen uptake than simple DME. The reaction of chemically generated superoxide with this electrolyte shows that almost no decomposition products (*e.g.*, lithium formate) are generated. This new electrolyte enables stable interfacial reaction, which in turn decreases the discharge voltage platform of LOBs (Fig. 16b and c). Huang *et al.*^177^ reported a methylated cyclic ether solvent 2,2,4,4,5,5-hexamethyl-1,3-dioxolane (HMD) for LOBs. This molecule does not contain any hydrogen atom on the α-carbon of the ether, thus avoiding hydrogen abstraction reactions. Therefore, the electrolyte using this solvent shows excellent stability in the presence of superoxide or singlet oxygen (Fig. 16d and e). In addition, sulfamide and sulfonamide-based molecules were proposed by Feng *et al.*^178^ This type of compound is capable of dissolving a reasonably high concentration of Li salts and is exceptionally stable under the harsh chemical and electrochemical conditions of LOBs. Particularly, *N*,*N*-dimethyl-trifluoromethanesulfonamide was found to be highly resistant to chemical degradation by peroxide and superoxide and thus enhanced the cycling stability of LOBs (Fig. 16f). Qin *et al.*^179^ introduced a “Solvent-in-Anion” strategy and synthesized a highly donating anion to enhance the electron donicity of the electrolyte (Fig. 16g), thus enabling the conversion between potassium superoxide (KO_2_) and peroxide (K_2_O_2_). Such an anion can passivate the electrode and allow the full charging back of K_2_O_2_ through the solution-mediated pathway without electrocatalysts.

## 4. Conclusion and molecular design principles

In this review, we summarize the various types of novel molecules emerging within the electrolyte of alkali metal batteries (selected representative molecular structures shown in Table 2) and discuss the underlying mechanism behind the improved electrochemical performance. Firstly, a brief analysis about the interfacial failure mechanism in alkali metal batteries is made. The aging mechanisms can be divided into three major categories: (i) the high reactivity of the prevailing electrolytes, (ii) the instability of SEIs/CEIs, and (iii) the notorious dendrites' growth. Secondly, we analyze the interfacial stability of alkali metal anodes when various novel molecules are used in the electrolytes, including ethers, carbonates, sulfonamides, phosphates, diluents and salts. Moreover, the electrolyte chemistry at the cathode is also discussed when these novel molecules are used. All in all, this review not only provides the latest development in electrolytes of alkali metal batteries but also sheds light on the further design of the electrolytes for high-performance alkali metal batteries.

**Table 2:** Selected representative molecular structures and corresponding cell performances related to AMBs

Molecular structure	Electrolyte	Battery	Cathode	Capacity/capacity retention	Anode	Ref.
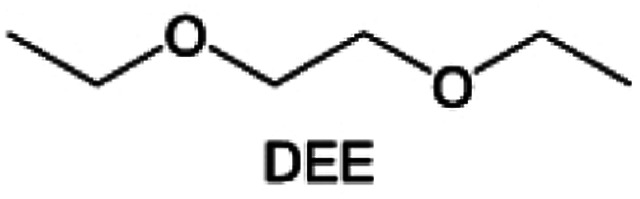	4 M LiFSI DEE	Li‖NMC811	4.8 mA h cm^−2^/4.4 V/1.3 mA cm^−2^	80% (182 cycles)	∼99.25% (150 cycles, 0.5 mA cm^−2^)	19
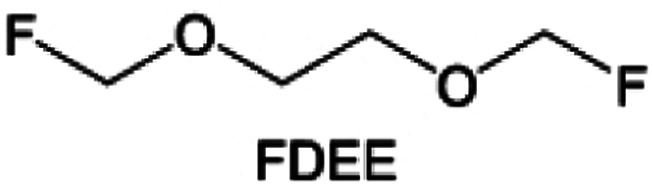	LiFSI : FDEE : TTE = 1 : 1.6 : 3	Li‖NMC811	1.7 mA h cm^−2^/4.6 V/C/3	95.9% (150 cycles)	∼99.4% (100–350 cycles, 0.5 mA cm^−2^)	54
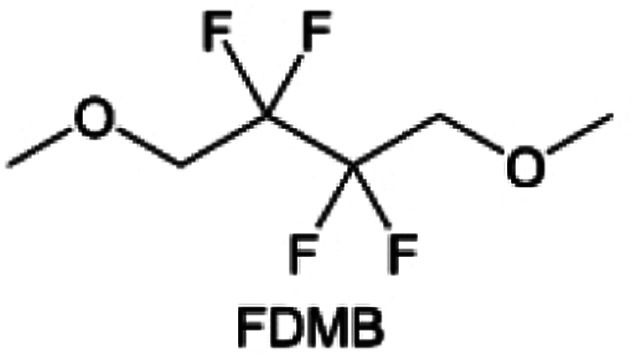	1 M LiFSI FDMB	Li‖NMC532	10 mA h cm^−2^/4.2 V/C/3	90% (420 cycles)	∼99.3% (5–300 cycles, 0.5 mA cm^−2^)	78
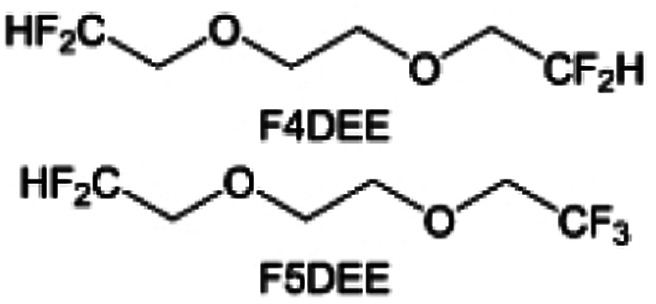	1.2 M LiFSI F4DEE/1.2 M LiFSI F5DEE	Li‖NMC811	4.9 mA h cm^−2^/4.4 V/0.3C	80% (200 cycles) 80% (180 cycles)	∼99.9% (100th–580th, 0.5 mA cm^−2^) ∼99.4% (100th–580th, 0.5 mA cm^−2^)	79
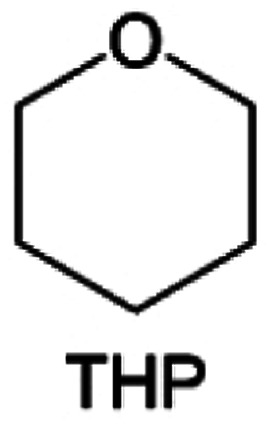	2 M LiFSI/0.4 M LiNO_3_ THP	Li‖NMC532	3 mA h cm^−2^/4.3 V/1 mA cm^−2^	CEs > 99.6% (100 cycles)	—	96
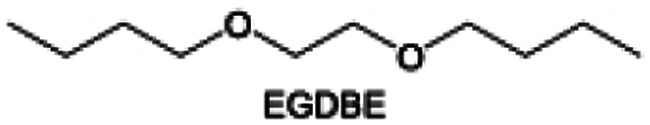	1 M LiFSI EGDBE : TTE = 1 : 1 (v/v)	Li‖NMC811	5 mg cm^−2^/0.5C	91.8% (300 cycles)	∼99.13% (250 cycles, 1 mA cm^−2^)	104
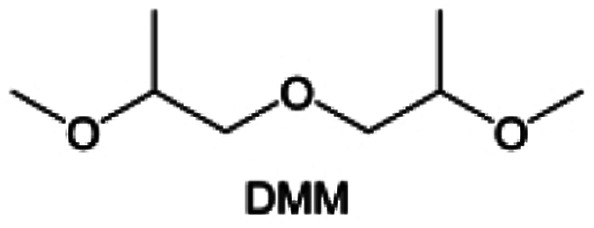	1 M KFSI-DMM	K‖PB	1 mg cm^−2^/4.2 V/100 mA g^−1^	98.9% (200 cycles)	98.66% (400 cycles, 0.2 mA cm^−2^)	105
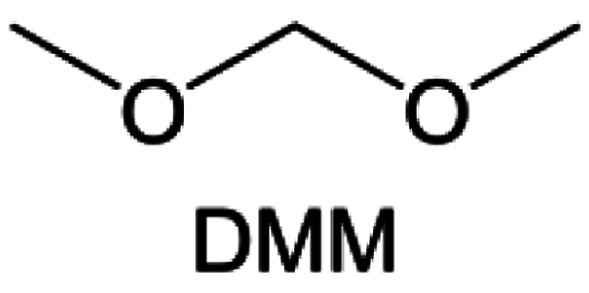	1 M LiFSI DMM	Li‖LTO	15.3 mg cm^−2^/0.5C	77.3% (200 cycles)	98.81% (100 cycles, 1 mA cm^−2^)	106
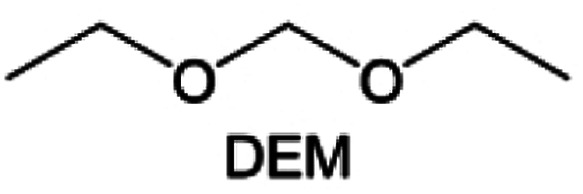	1 m LiFSI DEM	Li‖NMC811	2.5–3.5 mg cm^−2^/0.5C	100% (100 cycles)	∼99.1% (250 cycles, 1 mA cm^−2^)	107
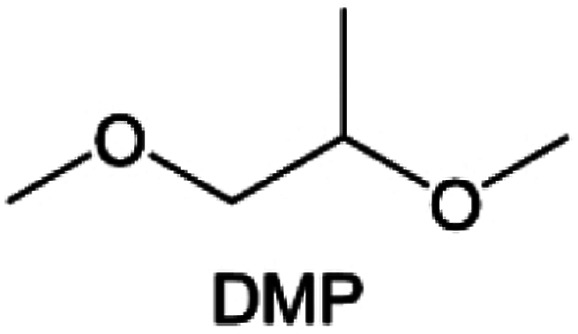	2 M LiFSI DMP	Li‖NMC811	11.4 mg cm^−2^/4.3 V/1C	86.0% (180 cycles)	∼99% (200 cycles, 0.5 mA cm^−2^)	108
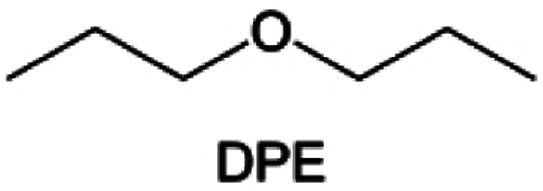	1.8 M LiFSI DPE	Li‖NMC811	1.6 mA h cm^−2^/4.3 V/1.6 mA cm^−2^	CE ∼99.92%	—	109
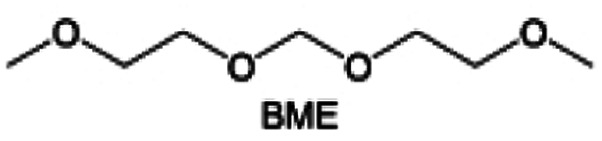	1 M LiFSI BME	Li‖LiFePO_4_	11 mg cm^−2^/4.0 V/1C	80% (1400 cycles)	—	110
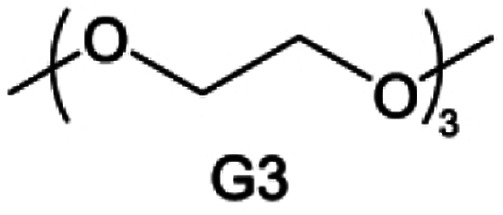	LiFSI : G3 : TTE = 1 : 1 : 3	Li‖NMC811	4 mA h cm^−2^/4.7 V/0.2C	80% (200 cycles)	∼99.2% (300 cycles, 0.5 mA cm^−2^, 1 mA h cm^−2^)	111
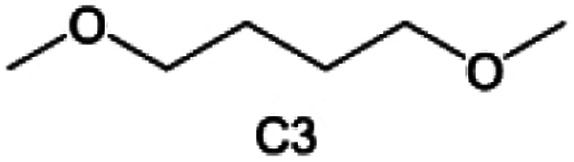	LiFSI : C3 : TTE = 1 : 1 : 3	Li‖NMC811	4 mA h cm^−2^/4.7 V/C/3	91.4% (100 cycles)	∼99.2% (300 cycles, 0.5 mA cm^−2^, 1 mA h cm^−2^)	112
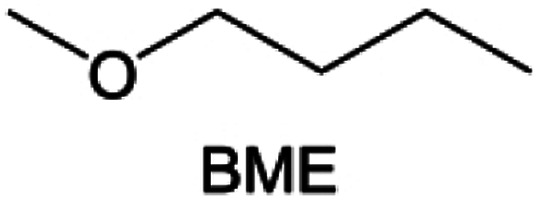	2 M LiFSI BME	Li‖SPAN	3.6 mA h cm^−2^/0.4C	83% (160 cycles)	99.1% (200 cycles, 1 mA cm^−2^)	113
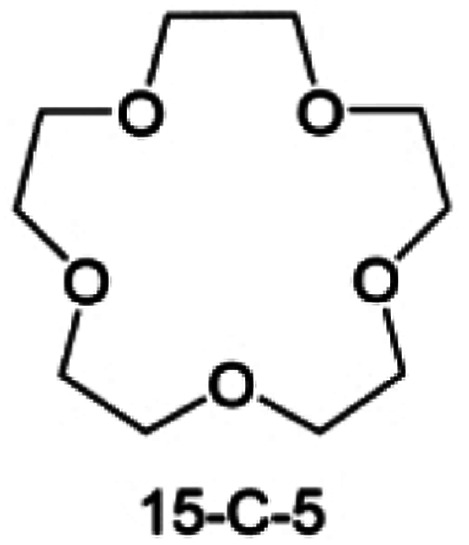	1 M LiPF_6_ EC : DMC = 1 : 1 (v/v) + 2% wt. 15-C-5	Li|NMC622	3.4 mg cm^−2^/0.5C	147 mA h g^−1^ (200 cycles)	100 cycles (0.5 mA cm^−2^, 1 mA h cm^−2^)	114
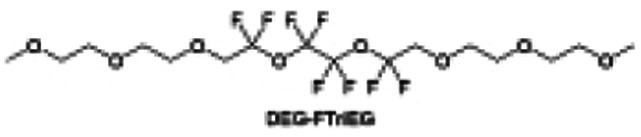	1 M LiFSA DEG-FTriEG	Li‖NMC811	0.9 mA h cm^−2^/4.4 V/0.2C	100 cycles	300 h (0.1 mA cm^−2^ 0.1 mA h cm^−2^)	115
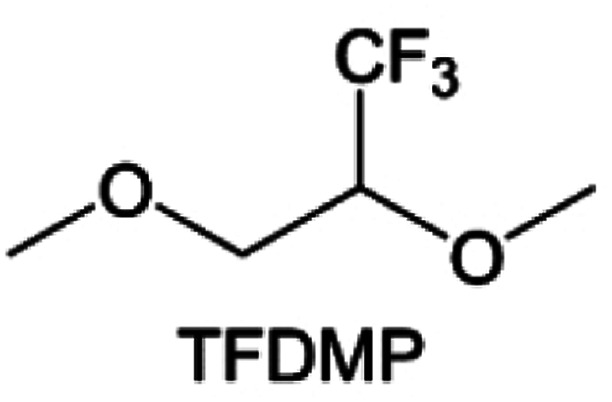	2 M LiFSI-TFDMP	Li‖NMC811	8 mg cm^−2^/1.6 mA cm^−2^	100% (2nd cycle to 451th cycle)	∼99.1% (450 cycle, 1 mA cm^−2^)	116
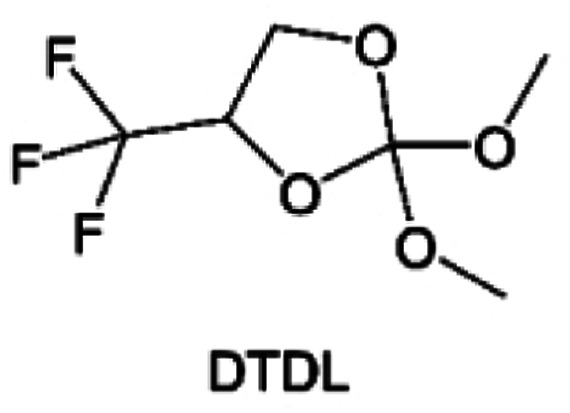	1 M/2 M LiFSI DTDL	Li‖NMC811	5 mg cm^−2^/4.3 V/0.5C	84% (200 cycles)	99.2% (at 250th, 0.5 mA cm^−2^)	117
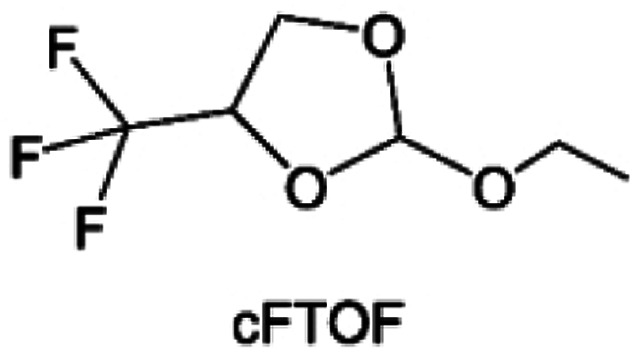	1 M LiFSI cFTOF	Li‖NMC811	5 mg cm^−2^/4.3 V/0.5C	100% (112 cycles)	97.8% (first 100 cycles) 99.0% (following 200 cycles)	118
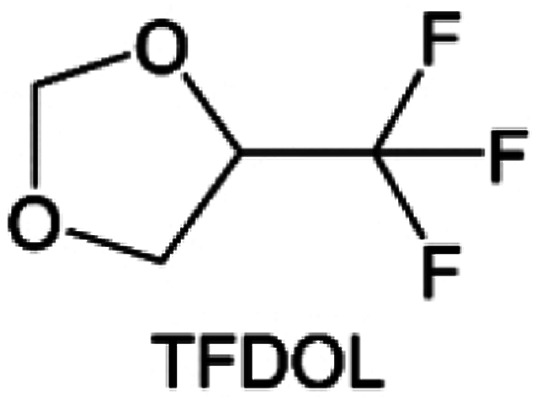	2 M LiFSI TFDOL	Li‖NMC811	8 mg cm^−2^/4.4 V/1C	89.0% (100 cycles)	∼98.5% (120 cycles, 1 mA cm^−2^)	119
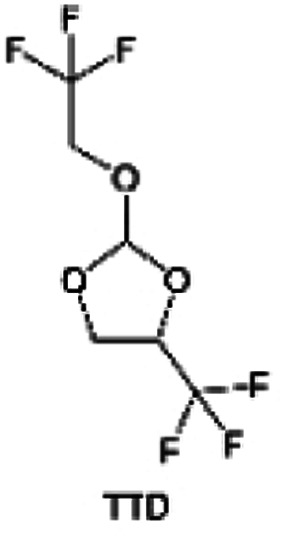	1.5 M LiFSI TTD : DME = 8 : 2 (v/v)	Li‖NMC811	8 mg cm^−2^/4.3 V/0.5C	75% (160 cycles)	∼99.3% (300 cycles, 2 mA cm^−2^)	120
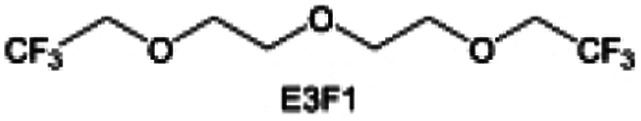	1 M LiFSA in E3F1	Li‖LiFePO_4_	1.81 mA h cm^−2^/3.8 V/C/3	250 cycles	700 h (1 mA cm^−2^, 1 mA h cm^−2^)	121
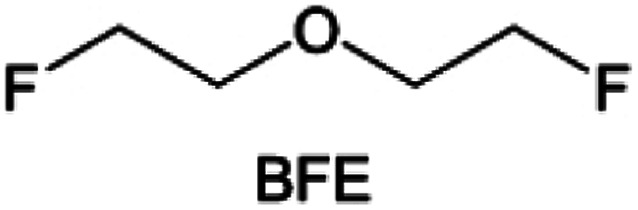	2 M LiFSI BFE	Li‖NMC811	3.5 mA h cm^−2^/4.4 V/7.0 mA cm^−2^	>90% (200 cycles)	∼99.75% (Aurbach's measurement)	122
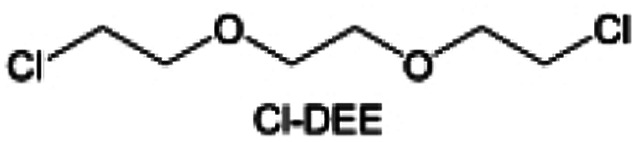	LiFSI : Cl-DEE : TTE = 1 : 1.6 : 3	Li‖NMC811	1.8 mA h m^−1^/4.5 V/C/3	95.44% (200 cycles)	∼99% (100 cycles)	123
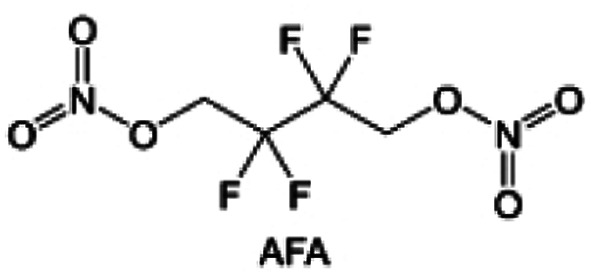	1 M LiTFSI DOL : DME = 1 : 1 (v/v) + 5 vol% AFA	Li‖S	4.0 mg cm^−2^/0.1C	50% (183 cycles)	—	124
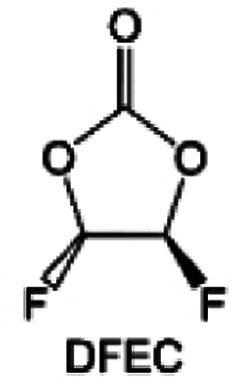	1.2 M LiPF_6_ DFEC : EMC = 3 : 7 (v/v)	Li‖NMC622	1.75 mA h cm^−2^/4.4 V/C/3	82% (400 cycles)	1000 h (2 mA cm^−2^)	127
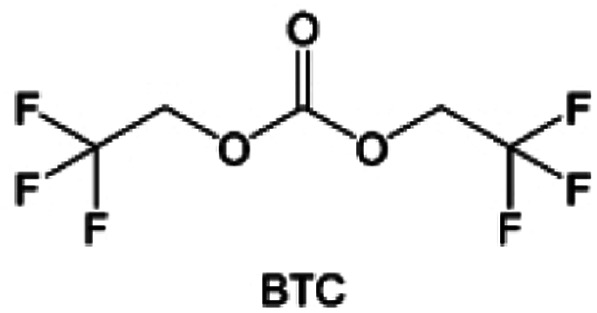	1 M LiPF_6_ FEC : BTC = 3 : 7 (v/v)	Li‖NMC811	8–8.5 mg cm^−2^/4.7 V/0.5C	95.1% (160 cycles)	98.8% (10–300 cycles, 0.5 mA cm^−2^)	129
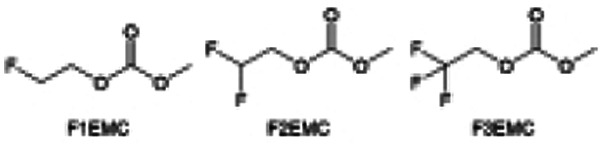	1 M LiPF_6_ FEC : F2EMC = 3 : 7 + 1 wt% LiDFP	Gr/SC-NMC811 (pouch cells)	4.4 V/1C	400 cycles	—	130
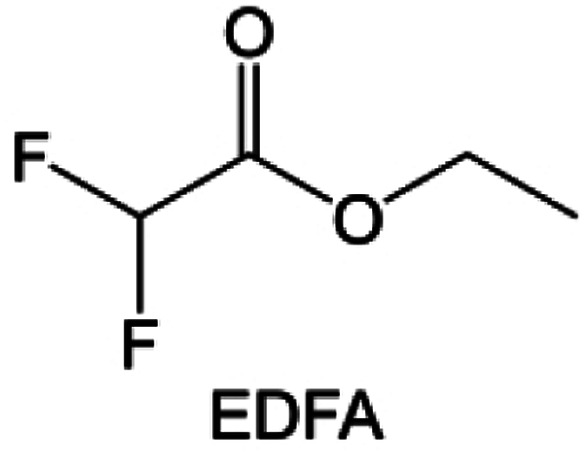	0.5 M LiFSI ETFA	NMC811‖Gr	Gr 3.3–4.0 mg cm^−2^/NMC811 4.6–6.0 mg cm^−2^/4.45 V/0.5C	80.5% (100 cycles)	Gr‖Li > 500 cycles (1–2.2 mg cm^−2^, 1C)	131
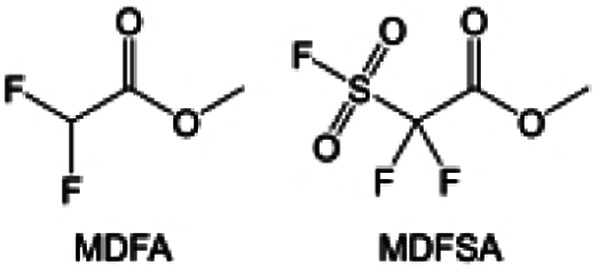	1 M LiTFSI MDFA : MDFSA : TTE = 4 : 1 : 5 (v/v/v)	Graphite‖NMC811	11.5 mg cm^−2^/4.5 V/0.5C	80.1% (400 cycles)	—	132
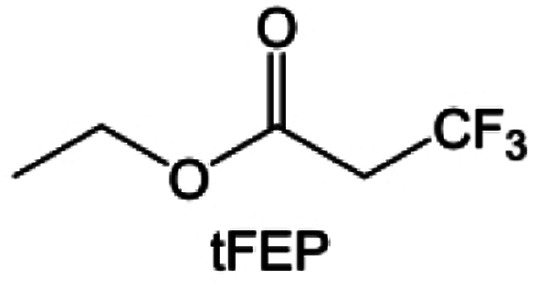	1 M LiBF_4_ + 1 M LiDFOB tFEP/FEC	Li‖NMC811	22 mg cm^−2^/4.6 V/0.5C	80.5% (100 cycles)	∼98.7% (100 cycles, 0.5 mA cm^−2^)	133
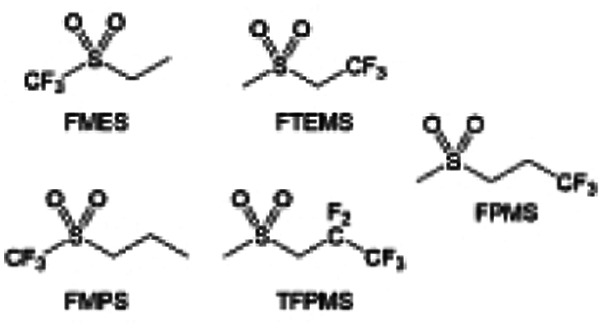	1.2 M LiPF_6_ FEC : TFPMS = 3 : 7 (v/v)	Graphite‖NMC622	7.94 mg cm^−2^/4.5 V/0.5C	81% (400 cycles)	—	134
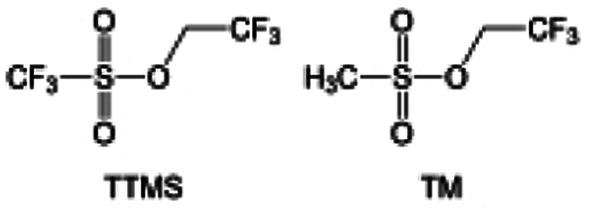	1.9 M LiFSI TTMS : TM = 1 : 2 (v/v)	Graphite‖LCO	(Graphite, 4.8 mg cm^−2^) 7.1 mg cm^−2^/4.55 V/2C	> 89% (5329 cycles)	—	135
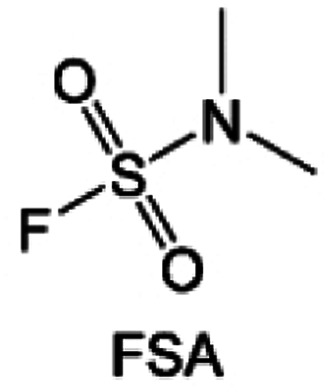	2.5 m LiFSI FSA	Li‖NMC622	1.6 mA h cm^−2^/C/3	89% (200 cycles)	99.03% (400 cycles, 0.5 mA cm^−2^)	136
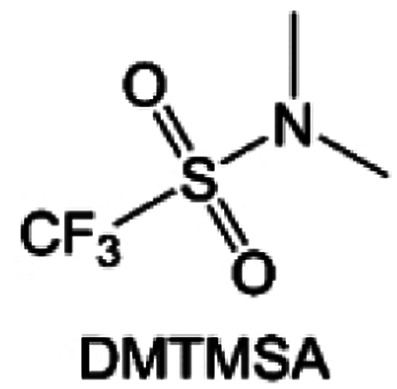	1 m LiFSI DMTMSA	Li‖NMC811	7.5 mg cm^−2^/4.7 V/0.5C	88.1% (100 cycles)	99% (345 cycles, 0.5 mA cm^−2^)	137
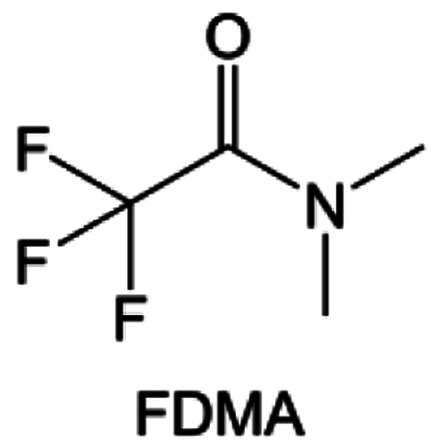	1 M LiTFSI FEC : FDMA = 1 : 1 (v/v)	Li‖NMC811	3.5 mA h cm^−2^/0.25C	88% (500 cycles)	∼99.3% (100 cycles, 1 mA cm^−2^)	138
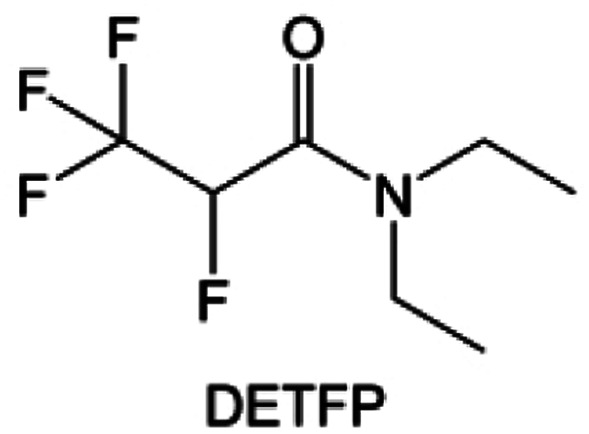	1 M LiPF_6_ EC : DEC = 1 : 1 (v/v) + 3.5% DETFP	Li‖NMC811	3 mg cm^−2^/1C	98.1% (100 cycles)	96.49% (Aurbach's measurement)	139
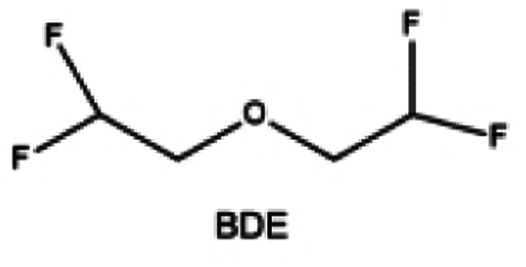	1.4 M LiFSI BDE/DME	Li‖LFP	2 mA h cm^−2^/3.8 V/1C	80% (500 cycles)	∼99.57% (Aurbach's measurement)	149
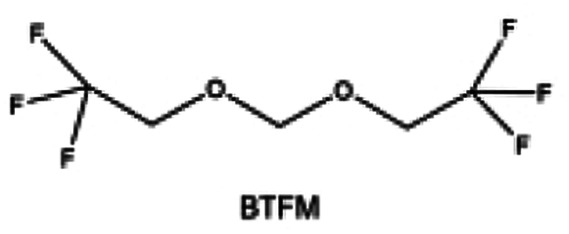	2 M LiFSI-3BTFM-1DME	Li‖NMC811	8 mg cm^−2^/4.4 V/3C	80% (596 cycles)	∼99.72% (Aurbach's measurement)	150
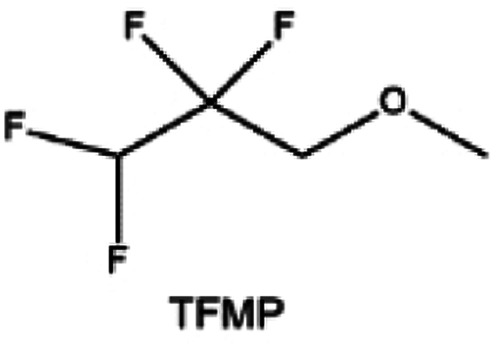	1 m LiFSI TFMP/DME	Li‖NMC811	3.5 mg cm^−2^/4.3 V/0.5C	95.3% (300 cycles)	∼99.6% (Aurbach's measurement)	151
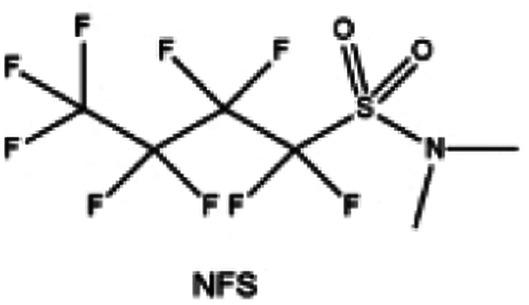	LiFSI : DME : NFS = 1 : 1 : 3	Li‖NMC811	3.4 mA h cm^−2^/4.4 V/0.5C	>90% (200 cycles)	∼99.5% (Aurbach's measurement)	152
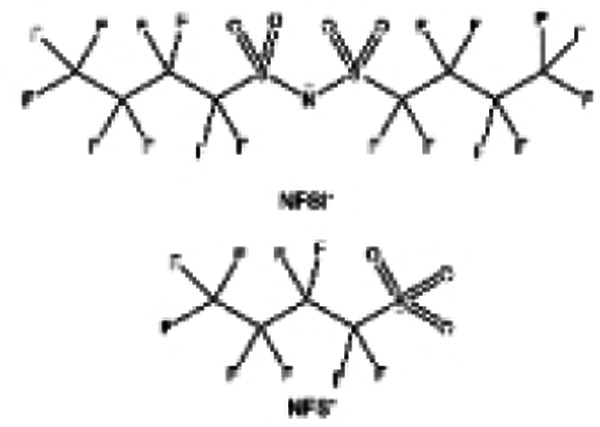	1 M LiNFSI DME 1 M LiNFS DME	Li‖NMC811	5 mg cm^−2^/4.4 V/C/3	98.5% CE (200 cycles) 96% CE (200 cycles)	—	157
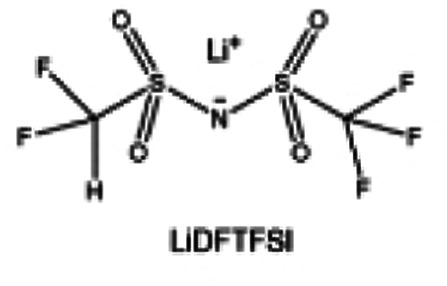	1 M LiDFTFSI EC-EMC (3 : 7, v/v)	Li‖NMC111	12.4 mg cm^−2^/4.2 V/0.2C	87% (200 cycles)	—	158
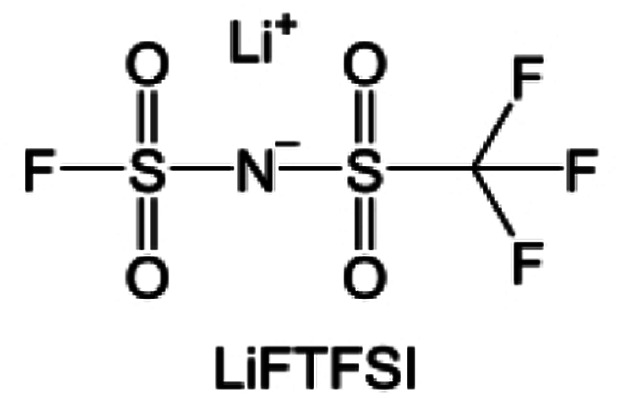	LiFTFSI PEO, EO : Li = 20 : 1	Li–S	0.9 to 1.1 mg cm^−2^/0.1C	800 mA h g_sulfur_^−1^ (60 cycles)	Li‖Li > 200 h (0.1 mA cm^−2^, 0.2 mA h cm^−2^)	160
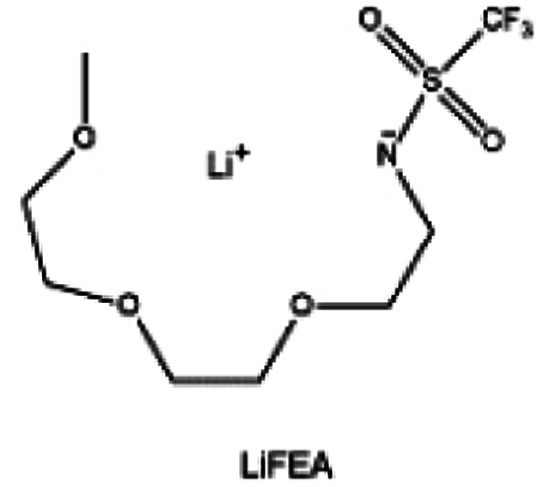	1 M LiPF_6_ EC/DEC (1 : 1, v/v) + 0.1 M LiFEA + 0.1 M LiNO_3_	Li‖NMC811	3 mg cm^−2^/4.3 V/5C	83.5% (500 cycles)	Li‖Li > 160 h (3 mA cm^−2^, 3 mA h cm^−2^)	162
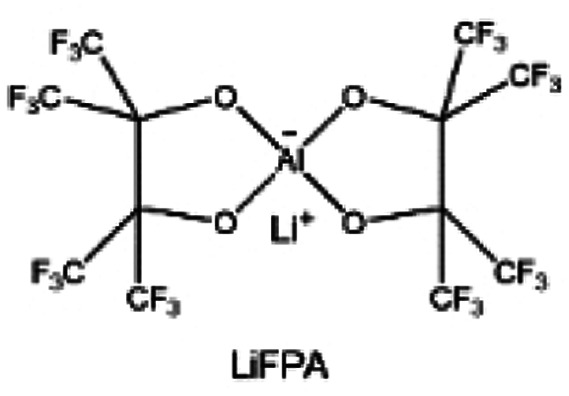	1 M LiFPA-EC/DMC	Li‖LiCoO_2_	1.65 mA h cm^−2^/4.3 V/0.2C	95.5% (150 cycles)	—	163
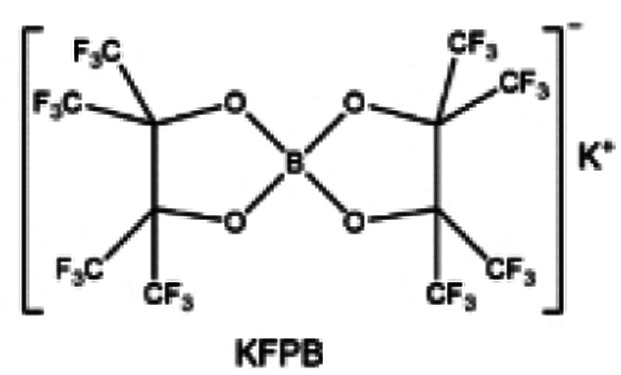	1 M LiPF_6_ EC/EMC (3 : 7, v/v) + 0.03 M KFPB	Li‖LiCoO_2_	1.7 mA h cm^−2^/4.4 V/0.2C	94% (500 cycles)	∼97.5% (Aurbach's measurement)	165
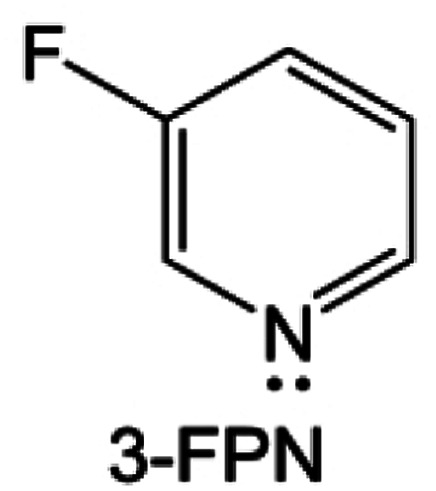	1 M LiTFSI 3-PFN	Li–S	2.5 mg_sulfur_ cm^−2^/0.1C	70.7% (50 cycles)	Li‖Li (750 h, 0.5 mA cm^−2^)	171
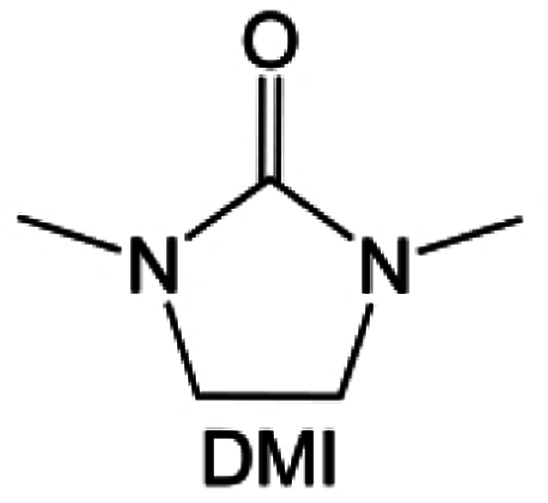	1 M LiTFSI + 0.5 M LiNO_3_ DMI	Li–S	5 mg_sulfur_ cm^−2^/0.03C	59.6% (80 cycles)	Li‖Li (215 cycles, 0.5 mA cm^−2^)	172
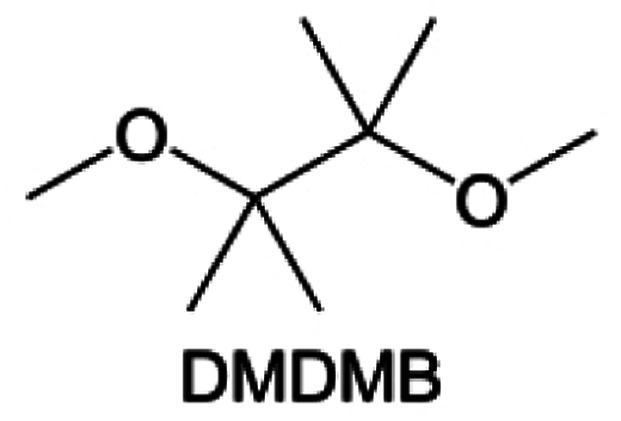	LiTFSI : DMDMB = 1 : 2	Li‖TiC (Li air)	50 μA cm^−2^	4.2 V (300 h)	—	176
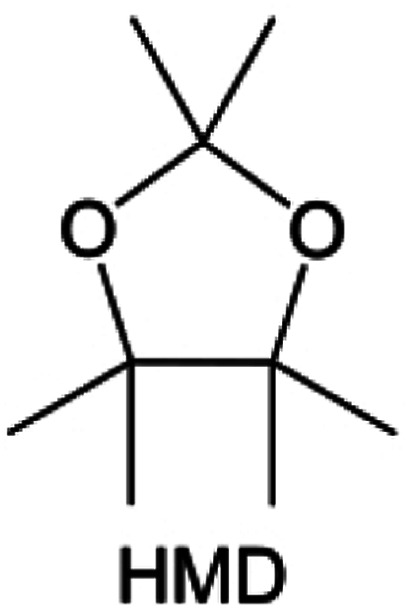	0.5 m LiTFSI + 20 mm boric acid (BA) HMD	Li‖carbon tube sponge	300 mA g^−1^	Without any cathode catalysts (157 cycles)	—	177
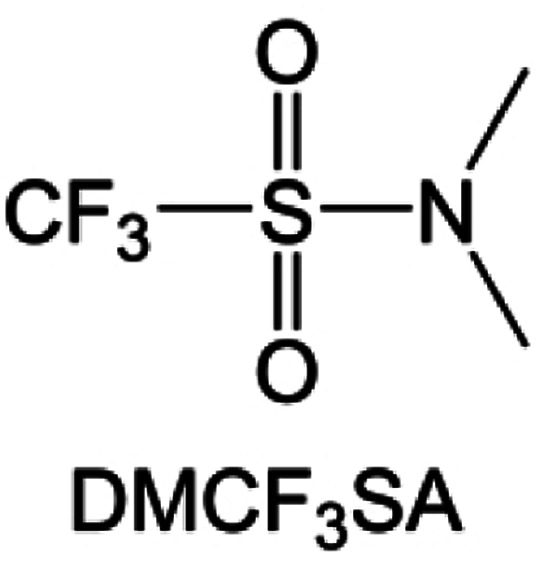	0.2 M LiTFSI DMCF_3_SA	Li‖CP-GDL	0.03 mA cm^−2^	(Electro)chemically stable (92 cycles)	—	178
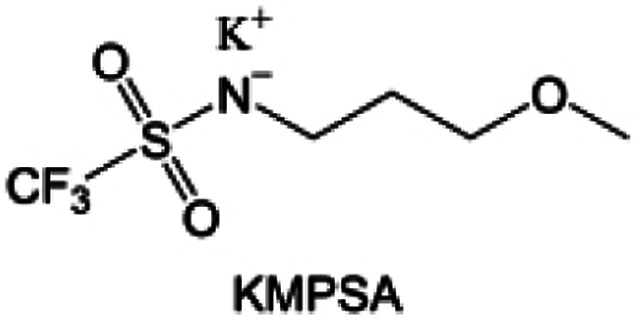	0.5 M KPF_6_ + 1.0 wt% KMPSA DME	K‖freestanding carbon paper with KO_2_ pre-deposition (0.2 mA h) (K–KO_2_)	49 mA g^−1^ KO_2_	84.4% round-trip energy efficiency (120 cycles)	—	179

Generally speaking, compared with the traditional electrolytes used in the alkali metal batteries, the electrochemical performances of the new electrolytes based on these novel molecules are all more or less enhanced. The improvement comes from various aspects, including the improved stability of the individual electrolyte component, unique solvation structures or stable interphases generated at the electrode/electrolyte interface. Although great progress has been made thanks to these novel molecular designs, the further development of practical alkali metal batteries still faces huge challenges.

(1) Molecular design and machine learning. The structure–property relationship between molecule structure of electrolyte, solvation structure, electrode–electrolyte interphases and electrochemical performance has not been clearly explained. Nowadays, instead of the design of new electrolytes mainly based on “trial and error” paradigm, which is time-consuming and inefficient, a new paradigm based on artificial intelligence or machine learning seems more interesting.

(2) Interface chemistry. With the significant advances in highly conductive solid-state electrolytes (SSEs) in recent years, the development of liquid or polymer electrolytes will be facing competition from SSEs. Considering the fact that electrolytes are thermodynamically stable to the Li metal anodes, all of the SSEs and liquid/polymer electrolytes would also face the interface issues in the battery. Only electrolytes with *in situ* formed passivation layers that could be self-healed can promise desired electrochemical performance for commercialization.

(3) Practical conditions. As the EV advances, the battery electrolytes must satisfy four requirements: high voltage, 5C fast charging, wide operation temperature range of −30 °C to +60 °C and nonflammability. Since higher energy is stored in the limited battery space, safety issues are becoming more prominent. Therefore, novel electrolytes in the EV batteries should be with improved safety, wider working temperature and voltage range and better rate capability.

(4) Market-driven. In most previous reports, electrochemical performance is always seen as “the acid test”. Cost and environmental issues during the production and usage process, which are the key factors that limit the commercial applications, are deliberately overlooked. In the long run, environmental and safety issues should be considered in the development of next-generation electrolytes.

(5) Differences in alkali metal anodes. The three alkali metals (Li, Na and K metals) not only all exhibit extremely high reactivity but also have significant physicochemical differences at the atomic level. A crucial aspect is the varied solubility of alkali metal salts with the same anion in electrolytes, a phenomenon influenced by the size of the cation. For example, different alkali metal fluorides (such as LiF, NaF and KF) may have different solubilities in the same electrolyte solution, primarily due to cation radius variations. This difference in solubility can significantly affect the physical stability of the SEI. Another critical factor is the diffusion of alkali metal ions within the SEI, which is also subject to the size effect of the cations. The larger ionic radius of Na and K compared to Li can lead to different diffusion characteristics within the SEI layer. This variability necessitates special attention in forming an SEI that not only provides a barrier to electron transfer but is also conducive to ion conduction. Tailoring the SEI structure and composition to facilitate the transport of larger Na^+^ and K^+^ while maintaining its protective properties is a challenging but essential task for improving the performance of Na and K batteries. Presently, there is a lack of consensus regarding the solubility differences of these alkali metal fluorides in contemporary electrolytes, and it is important to understand and optimize the transport of different cation ions within the SEI for enhancing the efficiency and longevity of alkali metal batteries.

## Author contributions

D. R. and Z. C. contributed equally to this work. Y. W. and X. R. structured this review. D. R., Z. C., J. F. and D. W. collected papers related to the topic and co-wrote the manuscript. The manuscript was revised by all authors.

## Conflicts of interest

There are no conflicts to declare.

## Supplementary Material
